# Use of Brazilian flora as the main source of new antimalarials: a systematic review

**DOI:** 10.1590/0074-02760240123

**Published:** 2025-06-02

**Authors:** Ana Rafaela Antunes Porto, Isabela de Brito Duval, Luisa Vitor Braga do Amaral, Izabela da Silva Oliveira, João Gabriel Acioli de Siqueira, Bruno Araújo de Albuquerque, Maria Alice Guarini Rocha, Gabriela Gomes Monteiro Lemos, Marcelo Eduardo Cardozo, José Bryan da Rocha Rihs, Ricardo Toshio Fujiwara, Ana Laura Grossi de Oliveira, Ramayana Morais de Medeiros Brito, Lilian Lacerda Bueno

**Affiliations:** 1 Universidade Federal de Minas Gerais, Instituto de Ciências Biológicas, Laboratório de Imunobiologia e Controle de Parasitos, Belo Horizonte, MG, Brasil; 2 Universidade Federal de Minas Gerais, Instituto de Ciências Biológicas, Programa de Pós-Graduação em Parasitologia, Belo Horizonte, MG, Brasil; 3 Universidade Federal de Minas Gerais, Faculdade de Medicina, Programa de Pós-Graduação em Ciências da Saúde: Infectologia e Medicina Tropical, Belo Horizonte, MG, Brasil

**Keywords:** malaria, Brazilian flora, natural compounds, antimalarial compounds

## Abstract

Plants represent an important source of compounds for treating malaria, highlighting the rich biodiversity of Brazilian flora as a vital resource for developing new, effective antimalarial drugs. The present study sought to shed light on the search for new compounds with antimalarial activity obtained from the Brazilian flora. In this sense, a systematic review was conducted using screening techniques based on “The Preferred Reporting Items for Systematic Reviews and Meta-Analysis” (PRISMA) protocol. Most of the plants collected in the studies were from the Amazon Rainforest, north of Brazil. Most of the isolated compounds were from the Apocynaceae family and the alkaloids were the main compounds isolated with significant antiplasmodial activity, followed by flavonoids and phenolic compounds. The Brazilian flora can source many compounds with potential antimalarial activity that can challenge *Plasmodium* drug resistance. However, new studies are still needed to elucidate the natural compounds activity for future application in Malaria treatment.

Malaria is an infectious disease found in tropical regions and caused by protozoan parasites of the *Plasmodium* genus. According to the most recent report on Malaria by the World Health Organization (WHO), about 249 million cases were registered worldwide in 2022; in the Region of Americas, over 480 000 cases were registered being distributed mainly across four countries: Venezuela (28%), Brazil (27%), Colombia (18%) and Peru (6%). In Brazil, more than 150,000 cases of the disease were reported in the same year.^1^ Although Brazil is taking ambitious measures to decrease the incidence and mortality rates of *P. falciparum* and *P. vivax* infections,^2^ eradicating the disease is challenging with the current resources. Factors such as poorly constructed shelters, insufficient funding for eradication programs, insecticide resistance, and most significantly, antimalarial drug resistance, persistently challenge the nation’s endeavours to decrease the rates of infection.^3^

*Plasmodium* resistance to chloroquine in the Americas was first reported between the 1960s and 1970s.^4^ Despite the use of other compounds like artemisinin and mefloquine, which are used in conjunction with chemotherapy, *P. falciparum* has exhibited resistance mechanisms.^5^ ^,^ ^6^ Therefore, ongoing research into new antimalarial drugs is crucial, with a significant focus on phytochemical studies.

Medicinal plants and their extracts have been employed for centuries by indigenous people from endemic areas, like the Brazilian Amazon forest. A promising approach for screening and testing novel antimalarial compounds involves relying on their accumulated ethnopharmacological knowledge.^7^ ^,^ ^8^ Historical records also provide compelling evidence of the efficacy of traditional medicinal plants. For instance, quinine and artemisinin, two of the most important antimalarial drugs, were isolated from plants used in traditional medicine - *Cinchona* spp. bark and *Artemisia annua* L. leaves and floral buds, respectively.^9^ ^,^ ^10^ These discoveries underscore the significant contributions of traditional knowledge to modern medicine. In the Brazilian context, the Apocynaceae family has been widely used by ethnic groups for treating fever and Malaria. The earliest records regarding the therapeutic use of this botanical family in Brazil date back to the 18th century, specifically for the treatment of intermittent fevers through the application of baths prepared with water resulting from boiling the bark of *Aspidosperma parvifolium*.^11^

Continued research and validation are necessary to integrate traditional knowledge with modern medicine effectively. In this sense, considering the great diversity found in Brazilian flora, the search for new compounds that can act as potential antimalarials has been strongly performed. In our systematic review, by combining ethnopharmacological insights with contemporary scientific methods, we focused on the state of the art in the search for new compounds obtained from native plants distributed across the biomes of Brazil, exhibiting biological activity against *Plasmodium* species, in both *in vitro* and *in vivo* assays. The results presented here shed light on the importance of Brazilian flora diversity, as an important source of chemical components that can act as promising antimalarial compounds.

Study conception

An accurate and extensive method was used to review studies on the efficacy of plant-based compounds with antimalarial activity using scientific evidence and a systematic approach. This review assesses the current scientific understanding of the measurable effects of plant-derived compounds and advances in scientific theory, laying the foundation for an evidence-based search for new promising effective antimalarials. In addition, this research thoroughly analyses the complex nature and biological activity of chemical molecules obtained from the Brazilian flora by utilising multiple sources of evidence, assessing their credibility, identifying gaps in the current literature, and providing perspectives for future research.

PROSPERO registration

This systematic review was conducted following the Preferred Reporting Items for Systematic Reviews and Meta-Analyses statement guidelines [Supplementary data (Table I)].^12^ The study protocol was registered in the International Prospective Register of Systematic Reviews (ID: CRD42023484094). The review question was: ‘What is known about the effectiveness of Brazilian plants with antimalarial potential?’ 

Search strategy and selection criteria

A comprehensive search on Pubmed (Medline), Embase, Scopus, Web of Science, Scielo, Biblioteca Virtual de Saúde (BVS/Lilacs), and Google Scholar databases was performed from June 2023 until October of the same year. The analysis included studies that disclosed all Brazilian flora plants used in the search for potential new antimalarials. The keywords were defined using the PICO acronym through a search utilising Medical Subject Headings (MeSH). The DeCS (LILACS) and Emtree (EMBASE) descriptors were adapted for each database. The search terms included “malaria”, “Brazil”, “plants”, “prevent”, and “treatment”, as stated in the search strategy (Supplementary data). Furthermore, we conducted a manual screening of the reference lists of the retrieved articles and relevant review studies to identify any potentially missed eligible trials. Articles were collected without any limitation on publication date or language.

The articles were exported in CSV/RIS format and uploaded on the Rayyan Software^®^. Two authors independently scanned the titles and abstracts of studies identified by the search. Whenever necessary, a third reviewer was consulted to resolve discrepancies. The researchers analysed full-text articles and screened each paper following the inclusion criteria. These criteria consisted of (a) articles that focused on Brazilian plants from native or adapted flora used to prevent or treat malaria, (b) original research and full-text available, and (c) papers that reported sufficient scientific data on the *in vivo* and/or *in vitro* experiments, as reduction of parasitaemia, half maximum inhibitory concentration (IC50), selectivity index (SI), 50% cytotoxic concentration (CC50), and other important results that characterised efficient prevention or treatment of the experimental infection. The classification of native and adapted plants in the Brazilian flora was determined through the deposit of specimens in the Reflora Virtual Herbarium administered by the Institute of Research Rio de Janeiro Botanical Garden. This effort was one main objective of the Global Strategy for Plant Conservation, operating under the Convention on Biological Diversity (https://reflora.jbrj.gov.br/reflora/). Publications unrelated to Malaria were excluded. Articles were selected and the relevant data were extracted into Excel spreadsheets.

Data extraction

The data collected included: (1) the article’s reference, popular name, species, and families of the plants, Brazilian biomes, and the score quality of the included studies; (2) active compounds families, and parts of the plants used in the studies; (3) *in vitro* data: *P. falciparum* strain, percentage of parasitaemia reduction, IC50, SI, CC50, and effectiveness results; (4) *in vivo* data: mice lineage, the dosage of the active principle/compound, blood smear, treatment regimen, percentage of parasitaemia reduction, and effectiveness results.

Quality grading

The quality of the individual studies according to the methodological scoring system, adapted from,^13^ was assessed independently by the authors, who used eight items to assess the quality of the studies: (1) In the study design, the sampling method (*in vivo* or *in vitro*) used is well described?; (2) in case of *in vivo* study, the source of the compounds are well described? (does not apply to *in vitro* studies); (3) in the case of *in vivo* study, the animal group size was adequate? In the case of *in vitro* study, how many repetitions were performed?; (4) the nature of the compounds or active molecules used in the assays are well described?; (5) the methods applied to determine the statistical significance of the results are described?; (6) the percentage of parasitaemia is provided?; (7) in case of *in vitro* studies, data such as IC50 and SI were provided?; (8) was it established a relationship between the dosage/concentration of the compounds/active molecules with the parasitaemia in the results? The studies were evaluated using a scoring system that ranges from 0 to 3 for low quality, 4 to 6 for moderate quality, and 7 to 8 for high quality.^13^

Literature search and selection criteria

From the specified electronic databases, a total of 325 articles were retrieved from the subsequent searches. After removing duplicate articles, the number of studies eligible for further evaluation was reduced to 193. Through a review of titles and abstracts based on predetermined inclusion and exclusion criteria, 98 studies were excluded. Subsequently, the full text of the remaining 73 articles was carefully examined, excluding 21 articles with reasons. Therefore, 52 records were included in this study (Fig. 1). The main characteristics of the papers are shown in Table. Thus, about 190 Brazilian plants were extensively studied for their antimalarial activity.

**Fig. 1:**
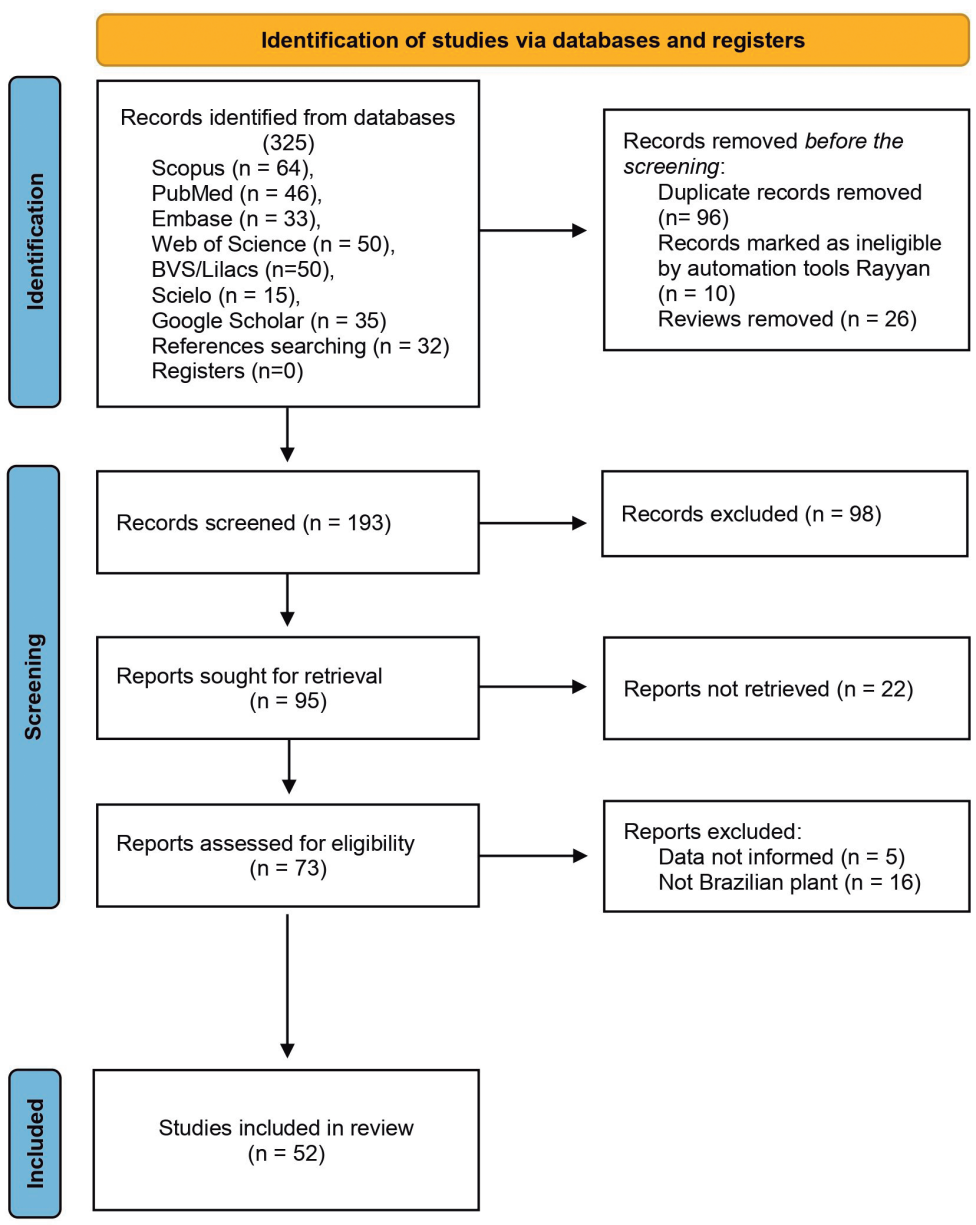
selection criteria. The selection of the studies was made based on two steps: (1) identification and (2) screening, after the exclusion of studies that did not fit the selection criteria, 52 articles were thoroughly analysed (3).

**TABLE:** The fifty-two included articles of Brazilian plants studied as potential antimalarials

N	Ref.	Journal	Impact factor	Title
1	^15^	Memórias do Instituto Oswaldo Cruz	2758 (2022)	*In vitro* inhibition of *Plasmodium falciparum* by substances isolated from Amazonian antimalarial plants
2	^16^	Antimicrobial Agents and Chemotherapy	4.9 (2022)	Antiplasmodial activity of Aryltetralone Lignans from *Holostylis reniformis*
3	^17^	Memórias do Instituto Oswaldo Cruz	2.747 (2022-2023)	*Aspidosperma* (Apocynaceae) plant cytotoxicity and activity towards malaria parasites. Part II: experimental studies with *Aspidosperma ramiflorum in vivo* and *in vitro*
4	^18^	Journal of Ethnopharmacology	5.195 (2022-2023)	Antiplasmodial and cytotoxic effects of the methanol extract, canthinone alkaloids, squalene- and protolimonoid-type triterpenes from *Homalolepis suffruticosa* root
5	^19^	Journal of the Brazilian Chemical Society	2.135 (2022-2023)	Leaves from the tree *Poincianella pluviosa* as a renewable source of antiplasmodial compounds against Chloroquine-resistant *Plasmodium falciparum*
6	^20^	Malaria Journal	3.469 (2022-2023)	Anti-malarial activity of indole alkaloids isolated from *Aspidosperma olivaceum*
7	^21^	Journal of Ethnopharmacology	5.195 (2022-2023)	Investigation of plant extracts in traditional medicine of the Brazilian Cerrado against protozoans and yeasts
8	^22^	Phytomedicine	7.9 (2022-2023)	*In vitro* and *in vivo* antimalarial potential of oleoresin obtained from *Copaifera reticulata* Ducke (Fabaceae) in the Brazilian Amazon rainforest.
9	^23^	Malaria Journal	3.469 (2022-2023)	*Aspidosperma pyrifolium*, a medicinal plant from the Brazilian caatinga, displays a high antiplasmodial activity and low cytotoxicity
10	^24^	ACS Omega	3.7 (2022-2023)	Chemical genomic profiling unveils the *in vitro* and *in vivo* antiplasmodial mechanism of açaí (*Euterpe oleracea* Mart.) polyphenols
11	^25^	Malaria Journal	3.469 (2022-2023)	*In vitro* and *in vivo* assessment of the anti-malarial activity of *Caesalpinia pluviosa*
12	^45^	African Journal of Pharmacy and Pharmacology	3.765 (2022)	*In vivo* and *in vitro* evaluation of antiplasmodial activity of *Amasonia campestris* (Aubl.) Moldenke
13	^46^	Journal of Ethnopharmacology	5.195 (2022-2023)	Antimalarial activity of extracts and fractions from *Bidens pilosa* and other *Bidens* species (Asteraceae) correlated with the presence of acetylene and flavonoid compounds.
14	^47^	Phytomedicine: international journal of phytotherapy and phytopharmacology	6.656 (2022-2023)	*In vitro* antiplasmodial activity of extract and constituents from *Esenbeckia febrifuga*, a plant traditionally used to treat malaria in the Brazilian Amazon
15	^48^	Memórias do Instituto Oswaldo Cruz	2.747 (2022-2023)	*Aspidosperma* (Apocynaceae) plant cytotoxicity and activity towards malaria parasites. Part I: *Aspidosperma nitidum* (Benth) used as a remedy to treat fever and malaria in the Amazon
16	^49^	Memórias do Instituto Oswaldo Cruz	2.758 (2022)	Screening of the antimalarial activity of plants of the Cucurbitaceae family.
17	^50^	Journal of Ethnopharmacology	5.195 (2022-2023)	Screening for antimalarial activity in the genus *Plothomorphe*
18	^51^	Journal of Ethnopharmacology	5.195 (2022-2023)	Antimalarial activity of *Cinchona*-like plants used to treat fever and malaria in Brazil
19	^52^	Phytotherapy research: PTR	6.388 (2022-2023)	Antimalarial activity of *Bidens pilosa* L. (Asteraceae) ethanol extracts from wild plants collected in various localities or plants cultivated in humus soil
20	^53^	Pharmaceutical Biology	3.889 (2022-2023)	Effect of piplartine and cinnamides on *Leishmania amazonensis, Plasmodium falciparum* and on peritoneal cells of Swiss mice
21	^54^	Pharmacognosy Magazine	0.7(2022-2023)	Evaluation of biological activity, toxicity, and phytochemical content of *Bowdichia virgilioides* (Fabaceae) aqueous extract
22	^55^	Journal of Herbal Medicine	2.542 (2023)	Antiplasmodial activity of hydroalcoholic extract from Jucá (*Libidibia ferrea*) pods
23	^56^	Pathogens	4.532 (2022-2023)	Chemical composition and *in vitro* antiplasmodial activity of the ethanolic extract of *Cyperus articulatus var. nodosus* residue
24	^57^	Food Research International	7.425 (2023)	Camu-camu (*Myrciaria dubia*) seeds as a novel source of bioactive compounds with promising antimalarial and antischistosomicidal properties
25	^58^	Brazilian Journal of Medical and Biological Research	2.904 (2023)	Antimalarial activity of crude extracts from Brazilian plants studied *in vivo* in *Plasmodium berghei*-infected mice and *in vitro* against *Plasmodium falciparum* in culture
26	^59^	Parasitology Research	2.383 (2023)	Antimalarial potential of leaves of *Chenopodium ambrosioides* L.
27	^60^	Malaria Journal	3.469 (2022-2023)	Two series of new semisynthetic triterpene derivatives: differences in anti-malarial activity, cytotoxicity and mechanism of action
28	^61^	Journal of Ethnopharmacology	5.195 (2022-2023)	*In vitro* antiplasmodial activity of Brazilian Cerrado plants used as traditional remedies
29	^62^	Annals of the Brazilian Academy of Sciences	1.813 (2022-2023)	*In vitro* antimalarial activity of six *Aspidosperma* species from the state of Minas Gerais (Brazil)
30	^63^	Malaria Journal	3.469 (2022-2023)	*Aspidosperma* species as sources of anti-malarials: uleine is the major anti-malarial indole alkaloid from *Aspidosperma parvifolium* (Apocynaceae)
31	^64^	Malaria Journal	3.469 (2022-2023)	*In vitro* and *in vivo* anti-malarial activity of plants from the Brazilian Amazon.
32	^65^	Journal of Ethnopharmacology	5.195 (2022-2023)	Antiplasmodial activity of the andiroba (*Carapa guianensis* Aubl., Meliaceae) oil and its limonoid-rich fraction
33	^66^	Phytomedicine: international journal of phytotherapy and phytopharmacology	6.656 (2022-2023)	Antiplasmodial activity of *Apidosperma indole* alkaloids
34	^67^	Planta Medica	2.7 (2022)	*In Vitro* and *in vivo* antimalarial activity of essential oils and chemical components from three medicinal plants found in Northeastern Brazil
35	^68^	Journal of Ethnopharmacology	5.195 (2022-2023)	*In vitro* antiplasmodial activity and identification, using tandem LC-MS, of alkaloids from *Aspidosperma excelsum*, a plant used to treat malaria in Amazonia
36	^69^	Journal of Ethnopharmacology	5.195 (2023)	New evidences of antimalarial activity of *Bidens pilosa* roots extract correlated with polyacetylene and flavonoids
37	^70^	Journal of Ethnopharmacology	5.195 (2022-2023)	Ethnopharmacological evaluation of medicinal plants used against malaria by quilombola communities from Oriximiná, Brazil
38	^71^	Malaria Journal	3.469 (2022-2023)	*In vitro* and *in vivo* anti-malarial activity of limonoids isolated from the residual seed biomass from *Carapa guianensis* (andiroba) oil production
39	^72^	Natural Product Research	2.488 (2022-2023)	*In vivo* antimalarial efficacy of acetogenins, alkaloids and flavonoids enriched fractions from *Annona crassiflora* Mart.
40	^73^	European Journal of Medicinal Chemistry	7.088 (2023)	New antimalarial and cytotoxic 4-nerolidylcatechol derivatives
41	^74^	Phytotherapy Research	7.2 (2022-2023)	*In vivo* and *in vitro* antimalarial activity of 4-Nerolidylcatechol
42	^75^	ACS publications	16.383 (2022-2023)	Antimalarial activity of physalins B, D, F, and G
43	^76^	Journal of Chromatography A	4.601 (2022-2023)	Isolation of secondary metabolites from *Hortia oreadica* (Rutaceae) leaves through high-speed counter-current chromatography
44	^77^	Memórias do Instituto Oswaldo Cruz	2.747 (2022-2023)	*In vitro* and *in vivo* antimalarial activity and cytotoxicity of extracts, fractions and a substance isolated from the Amazonian plant *Tachia grandiflora* (Gentianaceae)
45	^78^	Acta Amazonica	1.091 (2022-2023)	*In vitro* and *in vivo* antimalarial activity of the volatile oil of *Cyperus articulatus* (Cyperaceae)
46	^79^	Parasitology Research	2.383 (2023)	In silico evaluation and *in vitro* growth inhibition of *Plasmodium falciparum* by natural amides and synthetic analogs
47	^80^	Molecules	4.927 (2023)	Chemical composition of *Aspidosperma ulei* Markgr. and antiplasmodial activity of selected indole alkaloids
48	^81^	Drug Develpment Research	5.004 (2023)	Antimalarial activity of compounds and mixed fractions of *Cecropia pachystachya*
49	^82^	Revista da Sociedade Brasileira de Medicina Tropical	2.141 (2022-2023)	Efeito de *Momordica charantia* L. em camundongos infectados por *Plasmodium berghei*
50	^83^	Malaria Journal	3.469 (2022-2023)	Anti-malarial activity and toxicity assessment of *Himatanthus articulatus*, a plant used to treat malaria in the Brazilian Amazon
51	^84^	Medicinal Chemistry Research	2.351 (2023)	Naphthoquinones isolated from *Eleutherine plicata* herb: *in vitro* antimalarial activity and molecular modeling to investigate their binding modes
52	^85^	International Journal of Herbal Medicine	2.2 (2022-2023)	Isolation of caryatin as an Antiplasmodial component of *Symphyopappus casarettoi* (Asteraceae)

Antimalarial compounds research in Brazil: an overview

Out of 52 studies included in this review, the majority were published by institutions from the Southeast region of Brazil with 24 studies (46.15%), followed by the North region, with 17 (32.69%) published articles. The Northeast, South, and Central-West regions published five (9.61%), three (5.76%) and two (3.84%) studies, respectively (Fig. 2). At last, one study (2%) was published by French authors [Supplementary data (Table II)].

**Fig. 2:**
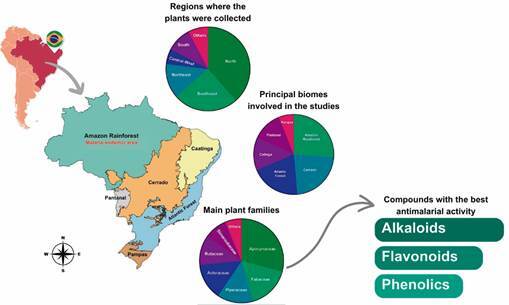
geographical and botanical analysis of plants studied for antimalarial activity in Brazil. The map highlights Brazil’s biomes and malaria-endemic regions, focusing on the Amazon Rainforest. Pie charts summarise (i) the regions where plants were collected, with the North contributing the majority, (ii) the principal biomes involved in the studies, with the Amazon Rainforest and cerrado dominating, and (iii) the main plant families studied. The compounds with the best antimalarial activity identified in these studies are alkaloids, flavonoids, and phenolics.

In regards to the region of Brazil where the plants were collected to conduct each study, the North region emerges as the most prevalent, appearing within 20 (38.46%) studies, indicating a significant focus on plant collection in this area. The Southeast, while being the primary region for study conduction, contributes to a lesser extent to the plant collection, appearing in only 13 (25%) studies. The Northeast, the Central-West, and the South regions contributed to seven (13.46%), three (5.76%) and five (9.61%) studies, respectively. Furthermore, in four (7.69%) studies, the collection region was not specified [Supplementary data (Table II)].

Analysing the biomes of the studied plants [Fig. 2, Supplementary data (Table II)], the Amazon Rainforest emerges as the most investigated area, found in 39 of 52 studies (73.5%). The cerrado is also extensively explored, with 34 (65.38%) studies, followed by the Atlantic Forest, with 30 (57.69%). Other biomes include the caatinga, the Pantanal, and the pampas, with 20 (38.46%), 18 (34.61%), and nine studies (17.30%), respectively.

A wide taxonomic distribution of plant families was found (Fig. 2). Apocynaceae emerges as the predominant family, featuring in 12 (23.07%) studies. Followed by Fabaceae, in seven (13.46%) studies, and Piperaceae and Asteraceae in six (11.53%) studies each. Other families include Rutaceae in five studies (9.61%), Simaroubaceae in four studies (7.69%) and Anacardiaceae, Gentianaceae and Euphorbiaceae in three studies (5.76%) each. Additionally, Annonaceae, Meliaceae, Cucurbitaceae, Rubiaceae, Sapindaceae, and Cyperaceae are each represented in two studies (3.84%); and the families Arecaceae, Loganiaceae, Aristolochiaceae, Flacourtiaceae, Calophyllaceae, Ebenaceae, Clusiaceae, Urticaceae, Solanaceae, Verbanaceae, Platanaceae, Rosaceae, Poaceae, Melastomataceae, Malpighiaceae, Lecitidacea, Rhamnaceae, Convolvulaceae, Humiriaceae, Amaranthaceae, Lamiaceae, Myrtaceae, and Iridaceae, each appeared in a single study (1.92%) [Supplementary data (Table II)].

Plant extracts and compounds: source and chemical composition

Concerning the plant material subjected to extraction, leaves and bark are the most frequently investigated parts in 20 (38.46%) articles, followed closely by roots in 19 (36.53%). Stems are also a significant focus, appearing in 10 (19.23%) studies. Seeds are used in six (11.53%) studies, and various components of fruits, including peel, whole fruit, and pulp, are scrutinised in four (7.69%). Whole or aerial plant extraction are employed in three (6.1%) studies, while trunks and branches are each featured in three studies (5.76%). Rhizomes are mentioned in two studies (3.84%), and other plant-specific and miscellaneous parts encompass four studies (7.69%) [Supplementary data (Table III)].

The classes of bioactive compounds extracted from these plants reveal a diverse chemical landscape [Supplementary data (Table III)]. Alkaloids predominate, featuring in 26 (50%) studies. Within alkaloids, monoterpenes are present in eight (15.38%) studies, sesquiterpene in four (7.69%), and triterpene alkaloids in six (11.53%). Other notable compound classes include flavonoids in eight (15.38%) and phenolics in seven (13.46%), with the last including subcategories such as tannins in four (7.69%), ellagic acid in three (5.76%), and anthocyanins in two (3.84%). Limonoids were identified in four studies (7.69%), 4-Nerolidylcatechol (4-NC) in two studies (3.84%), and polyacetylenes in two studies (3.84%). Additionally, six (11.53%) articles analysed compounds that do not belong to any of these families, and nine (17.30%) did not conduct any characterisation of the chemical compounds present in the studied plants [Supplementary data (Table III)].

Analysis of antiplasmodial activity of plants-derived compounds

A total of 46 out of 52 selected studies showed *in vitro* data. These studies displayed a variety of *P. falciparum* strains, with the chloroquine-resistant W2 strain being predominantly featured in 25 (54.34%) of the *in vitro* studies, followed by the *P. falciparum* chloroquine-sensitive 3D7 strain in 13 (28.26%) papers; the K1 strain appeared in eight (17.39%) papers, the BHz strain in two (4.34%) papers and the FcB1 strain in two (4.34%) papers. Five studies included strains not present elsewhere (10.86%) and three lacked specifications of which strain was used (6.52%) [Supplementary data (Table IV)].

The studies included in this review encompassed a variety of approaches in which plant-derived compounds were tested for antiplasmodial activity *in vitro*. Crude extracts were a predominant focus, featuring in 25 papers (54.34%). Isolated compounds were also extensively studied, appearing in 24 (52.17%) studies, followed by extract fractions in 13 (28.26%) and oils in four (8.69%) studies [Supplementary data (Table IV)].

The methodologies employed in these studies to measure parasitaemia levels and calculate IC50 also varied. The direct visualisation and counting of parasites through Giemsa stain was the most frequently applied technique, employed in 26 papers (56.52%). Biomolecules produced during *Plasmodium* sp. development were used to quantify parasitaemia reduction through the histidine-rich protein 2 (HRPII) assay and parasitic lactate dehydrogenase (pLDH) assay in seven (15.21%) and four (8.69%) studies, respectively. The (3H)-hypoxanthine uptake assay, utilised in 13 (28.26%) studies, allows to assess parasitaemia reduction by measuring the incorporation of radioactive hydrogen present in the hypoxanthine reagent into the parasite’s nucleic acids.^14^ Molecular biology was a less prevalent method, appearing in only two (4.34%) studies, and one (2.17%) did not disclose its methods.

In terms of data presentation, the focus predominantly centred on IC50 values, with all 43 *in vitro* studies providing this critical information. Furthermore, 22 studies (47.82%) included the presentation of the selectivity index (SI), while eight (17.39%) showed data that can allow the readers to calculate it [Supplementary data (Table IV)].

The best IC50 value observed was for neosergeolide, which is 0,001 μg/mL, and this compound is a quassinoid that was extracted from *Picrolemma sprucei*, a Simaroubaceae plant [Supplementary data (Table II)], by Andrade-Neto et al.^15^ Additionally, Andrade-Neto et al.^15^ also identified an alkaloid called aspidocarpine in the *Aspidosperma desmanthum*, species from the Apocynaceae family [Supplementary data (Table II)], with IC50 = 0,007 μg/mL, and one named ellipticine in *Aspidosperma vargasii*, also from the Apocynaceae family [Supplementary data (Table II)], with IC50 = 0,018 μg/mL. The compounds (7′R,8S,8′R)-4,5-Dimethoxy-3′,4′-methylenodioxy-2,7′-cyclolignan-7-on and 7′R,8S,8′S)-3′,4,4′,5-Tetramethoxy-2,7′-cyclolignan-7-one are lignans isolated from *Holostylis reniformis*, from the Aristolochiaceae family [Supplementary data (Table II)], with IC values of 0.26 ± 0.08 μg/mL and 0.63 ± 0.20 μg/mL, respectively.^16^ Finally, Andrade-Neto et al.^15^ reported a compound extracted from *Pothomorphe peltata* (Piperaceae) [Supplementary data (Table II)], called 4-nerolidylcatechol with an IC50 of 0.21 µg/mL. Aguiar et al.^17^ reported isositsirikine, a monoterpenoid indole alkaloid isolated from *Aspidosperma ramiflorum*, an Apocynaceae plant [Supplementary data (Table II)], whose IC50 is 0.2 ± 0.0 μg/mL, and the SI value was 83 with BGM cell line, and 113 with the HepG2 one. A good IC50 value (0.0548 ± 0.008 μg/mL) was also observed with 5-methoxycanthin-6-one by Boeno et al.,^18^ an alkaloid extracted from the species *Homalolepis suffruticosa* of the Simaroubaceae family [Supplementary data (Table II)] with a SI of 11,31. Ellagic acid, a phenolic compound isolated from the *Poincianella pluviosa* species of the Fabaceae family [Supplementary data (Table II)], showed an IC50 of 0.215 ± 0.007 µg/mL and SI > 4,651.1, using HepG2 cell line.^19^

Regarding the selectivity index, the best antiplasmodial activities were identified in *Poincianella pluviosa* (Fabaceae) by Souza et al.:^19^ ellagic acid, previously mentioned above; Fabaceae acetate extract, with an SI > 546.4 and IC50 of 1.83 µg/mL; and Fabaceae ethanol extract, which showed an SI > 145.3 and an IC50 = 50 6.88 ± 1.64 µg/mL. Additionally, a monoterpene extracted from the Apocynaceae family and a methanol extract from leaves of this family, had SI of 499.5 (Vero cells-*P. falciparum* W2) and 126, respectively, and IC50 of 7.0 ± 0.0 μg/mL and 7.2 ± 2.3 μg/mL, respectively.^20^ Albernaz et al.^21^ identified the ethyl acetate extract in *Diospyros hispida* (Ebenaceae) roots and the hexane extract in *Croton urucurana* (Euphorbiaceae) stem wood with SI and IC50 values of 435.8 and 1 ± 0.9 µg/mL, > 285.7 and 50 3.5 ± 0.02 µg/mL, respectively.

Parallel to this, 27 out of the 52 articles contemplated in this review reported *in vivo* data [Supplementary data (Table V)]. These studies displayed experimental models that employed a variety of *P*. *berghei* strains, with *Pb*-NK65 being the predominant strain, featured in 17 (62.96%) studies, followed by *Pb*-ANKA in four (14.81%) studies. An additional five (18.51%) studies utilised unspecified *P. berghei* strains, while one (3.70%) experimented with an unspecified strain of *P. chabaudi* [Supplementary data (Table V)].

The diversity of compound types in *in vivo* models is evident, with crude extracts constituting a significant focus, comprising 20 (74.07%) studies, while extract fractions were explored in 11 (40.74%). Isolated compounds featured in 10 (37.03%) studies, while oils were present in two (7.40%). Divergent dosage regimens were employed across studies. Dosages of ≥ 1000 were administered in six (22.22%) studies, doses between 500 and 999 were featured in eight (29.62%) studies, between 200 and 499 were administered in 14 (51.85%) studies, and lastly, dosages of ≤ 199 were prevalent in 15 (55.55%) studies [Supplementary data (Table V)].

The best *in vivo* result was by Souza et al.,^22^ with sesquiterpenes and diterpenes from the oleoresin of *Copaifera reticulata* from the Fabaceae family [Supplementary data (Table II)], administered orally, that reduced 96% of parasitaemia in mice infected with *P. berghei* at 200 mg/kg on day 11 and 93% at 100 mg/kg on day 11; this compound also showed low cytotoxicity. Ceravolo et al.^23^ showed that 93% of parasitaemia was reduced on day 5 by the oral administration of bark hydromethanolic fraction of the *A. parvifolium* species from the Apocynaceae family [Supplementary data (Table II)], which contained monoterpenes. Additionally, this same study showed a 79% reduction on day 5 with root-bark ethanol extract, 75% on day 5 with root ethanol extract, and 79% on day 5 with bark chloroform fraction. All these results were obtained with a dosage of 100 mg/kg and oral administration on Swiss mice infected with *P. berghei* NK65. Phenolics from *Euterpe oleracea*, included in the Arecaceae family [Supplementary data (Table II)], administered orally showed an 89,4 and 77,3% reduction of parasitaemia on days 6 and 7 with 20 mg/kg, 81 and 62,2% reduction at 15 mg/kg on days 6 and 7 against *P. chabaudi* on C57BL/6 mice.^24^ Kayano et al.^25^ also studied C57BL/6 mice infected with *P. chabaudi*, but they were treated orally with compounds from *Caesalpinia pluviosa* (Fabaceae) [Supplementary data (Table II)] and the 50% ethanolic fraction led to parasitaemia reduction of 79,4% and 72,1% at 50 mg/kg on days 6 and 7, respectively. Monoterpenes isolated from *Aspidosperma olivaceum*, of the Apocynaceae family [Supplementary data (Table II)] were used as an oral treatment for Swiss mice infected with *P. berghei* ANKA and the acidic fractions reduced parasitaemia by 79% at 100 mg/kg.^20^

Mice breeds employed in these studies exhibited small variability, and were dominated by Swiss outbred mice, featuring in 17 (62.96%) studies. Balb/c mice were utilised in six (22.22%) studies, followed by the C57BL/6 mice strain in one (3.70%) study [Supplementary data (Table V)]. The remaining three (11.11%) studies did not specify the mouse breed used. The duration of treatment exhibited a range, almost half (13, 48.14%) of studies employ Peter’s 4-day Suppressive Test.^24^ Studies employing 3-day treatment regimens constituted nine (33.33%) studies, and two (7.40%) employed an 8-day regimen. Uncommon treatment durations of five, seven, nine, and 10 days were less prevalent, with a single (3.70%) study each. Variability in the initiation of parasitaemia analysis was also observed. Day 5 emerged as the predominant time point, featuring in 16 (59.25%) studies. Day 4 and Day 11 were explored in four (14.81%) and two (7.40%) studies, respectively. The remaining days (1, 3, 6, 9, and 10) each featured in one (3.70%) study [Supplementary data (Table V)].

The historical study of malaria in Brazil shows the complexity and persistence of this disease that, as early as the nineteenth century, had a global distribution.^26^ With the arrival of colonisers in Brazil, came the forced migration of African slaves during the colonial era, a factor that contributed to the dissemination of the disease in the Brazilian territory. Here, the *Plasmodium* species found an ideal environment for its development, with anopheline vectors and favourable climate conditions.^27^ Since those times, there has been an ongoing public health initiative to promote measures aiming at the control of Malaria, including treatment strategies to minimise the vector infection rates and reduce transmission.

In Brazil, between 1905 and 1906, during efforts to control a Malaria epidemic, Carlos Chagas, an important Brazilian researcher, administered doses of quinine to railroad construction workers, an at-risk group for the infection. This event highlights que quinine’s status as the primary treatment for malaria in the early 20th century. However, the development of resistance by the parasite was first documented in 1907, in Rio de Janeiro.^27^

Moving to the second half of the 20th century, chloroquine use was widespread in Brazil. The Malaria Eradication Campaign (EMC), in 1957, introduced the medication into salt formulations, especially in areas where insecticide application was challenging, a strategy known as “Pinotti’s method”.^28^ Although this method ceased in Brazil by the 60s, it continued in other countries in the 70s, and chloroquine was still a key component of Malaria treatment. Nonetheless, *Plasmodium* species started to develop resistance to this medication in 1946, and by 1980 resistance was spread throughout the country.^29^

Therefore, *Plasmodium* resistance to the key medications in malaria treatment is a challenge for the control of the disease. Combining various therapies and exploring new compounds with antimalarial effects is of major importance. This research extends to natural compounds and, particularly in Brazil, where malaria is endemic to the Amazon region, urges the importance of traditional knowledge regarding natural compounds and the necessity of conducting further studies to assess their antiplasmodial activity. For that reason, the review conducted here highlights the significant contribution of Brazilian biodiversity to the field of antimalarial research, focusing on plant-derived compounds and their potential for therapeutic applications.

The dominance of the Apocynaceae, Fabaceae, and Piperaceae families in the reviewed studies reflects the abundance and rich chemical diversity, which underscores their relevance for future investigations. The predominant family of plants in the studies included in this review was Apocynaceae. These plants exhibit a worldwide distribution, from tropical to temperate climates, and various growth patterns.^30^ The Fabaceae family also deserves emphasis, with plants spread throughout the world and encompassing nearly 500 species with medical applications.^31^ Additionally, the Piperaceae family, with approximately 1000 species primarily found in tropical areas,^32^ emerged as the third most prominent family in the studies. These families encompass a wide range of bioactive compounds, including alkaloids, flavonoids, and phenolics, which exhibited promising antiplasmodial activity *in vitro* and *in vivo*. These three groups of compounds are secondary metabolites found in plants.^33^ ^,^ ^34^ Among these, alkaloids stood out due to their potent IC50 values, exemplified by neosergeolide, aspidocarpine, and ellipticine, which underscores the potential of these molecules for further research.

Alkaloids are characterised by nitrogen as its principal element and it is produced through various processes by different species, each with its unique chemical structure and specific functions. Plants naturally synthesise alkaloids as a defence mechanism against environmental adversities.^33^ Therefore, alkaloids have a wide range of applications based on their biological activity, such as analgesic properties,^35^ antimicrobial effects,^36^ insecticide properties,^37^ anti-inflammatory activity^38^ and, as it is the focus of this review, antimalarial effects.^33^

Phenolic compounds play a crucial role in controlling plant growth and regulating physiology, and they have many medical applications.^34^ They are a group of compounds that include, among others, flavonoids, phenolic acids, and tannins. Studies have shown the antimicrobial and antioxidant effects of these compounds.^39^ Flavonoids are secondary metabolites that occur in plants and are linked to biotic and abiotic stresses.^40^ They exhibit interesting biological activities, with medical applications being the most common to these compounds.^41^ For instance, researchers are exploring their anti-inflammatory activity,^42^ antitumoral effects,^43^ efficacy acting against the coronavirus infection,^44^ and antimalarials properties.^41^

In this review, Andrade-Neto et al^16^ and Costa et al^45^ have independently demonstrated the antiplasmodial activities of phenolic compounds and alkaloids *in vitro*, while Brandão et al^46^ showcased the efficacy of phenolic compounds against *Plasmodium*; Dolabela et al^47^ highlighted the alkaloid and flavonoids antiplasmodial activity, also *in vitro*. Additionally, Coutinho et al^48^ research revealed that alkaloid-rich fractions have antimalarial properties *in vivo*, just as Ferreira et al^24^ findings did with phenolic compounds.

Despite the promising findings, certain challenges remain. The lack of chemical characterisation in a significant proportion of studies and the limited reporting of selectivity indices point to the necessity of standardising methodologies in antimalarial research. Selectivity index is crucial for determining compound safety, and their consistent reporting would facilitate the comparison of bioactivity across studies. Furthermore, variability in experimental approaches, including parasitaemia measurement techniques, underscores the need for harmonised protocols to ensure reproducibility and comparability of results.

A critical aspect highlighted here was the exploration of how different classes of compounds performed against *Plasmodium* species with varying sensitivity to chloroquine (CQ). The analysis of antiplasmodial activity across plant-derived compounds highlights the potential to generate new insights into strain-specific activity profiles, particularly in the context of resistance to CQ. The review presented here encompassed in vitro studies that evaluated various *P. falciparum* strains, with notable emphasis on the CQ-resistant W2 strain, and the CQ-sensitive 3D7 strain. Among the findings, the quassinoid neosergeolide, extracted from *Picrolemma sprucei*, exhibited the most potent IC50 value of 0.001 µg/mL, indicating broad-spectrum activity that could be further explored for efficacy against resistant strains. Similarly, alkaloids such as aspidocarpine (IC50 = 0.007 µg/mL) and ellipticine (IC50 = 0.018 µg/mL), isolated from species of the *Aspidosperma* genus, demonstrated remarkable activity, suggesting their potential relevance in overcoming resistance. In contrast, phenolic compounds like ellagic acid (IC50 = 0.215 µg/mL) displayed more selective activity, with an exceptionally high selective index (SI) (SI > 4651.1) against CQ-resistant W2 strain. These findings underscores the potential of phenolics to maintain efficacy across resistance profiles while minimising cytotoxicity. Additionally, monoterpenes from *A. ramiflorum* demonstrated both significant activity (IC50 = 0.2 µg/mL) and SI (SI = 83 - 113), further supporting their potential as candidates for resistance-focused research.

On that account, the structural diversity of plant-derived compounds can be harnessed to address the challenge of drug resistance in *P. falciparum*. Compounds such as neosergeolide, aspidocarpine, and ellagic acid demonstrate the potential to generate strain-specific efficacy, while the activity of alkaloids and phenolics across resistant and sensitive strains can provide valuable insights into their mechanisms of action. Future studies should prioritise the isolation and structure elucidation of active compounds, complemented by detailed mechanistic studies to elucidate their mode of action. For instance, plant-derived compounds that disrupt parasite redox homeostasis, inhibit haemozoin formation, or interfere with other essential parasite pathways could serve as promising therapeutic targets.

Overall, this review reaffirms the significant potential of Brazilian plants as sources of antimalarial agents, and highlights the importance of a multidisciplinary approach to address the urgent need for new therapeutic tools in the face of rising *Plasmodium* resistance to existing drugs. By correlating compound classes with their efficacy against resistant and sensitive *Plasmodium* strains, researchers can uncover novel resistance-breaking compounds and expand the arsenal of tools available to combat malaria. Future research should combine high-throughput screening with advanced molecular techniques to elucidate the specific interactions between bioactive compounds and parasite targets, facilitating the translation of these findings into clinical interventions.
