# Supplementary material for: Use of Brazilian flora as the main source of new antimalarials: a systematic review
Source: Mem Inst Oswaldo Cruz. 2025 Jun 2;120:e240123. doi: 10.1590/0074-02760240123 (PMC12147452; doi:10.1590/0074-02760240123)
Supplement: Supplementary file 1 [file 1678-8060-mioc-120-e240123-s.pdf]

Search strategy

Scopus n = 64 - (malaria)AND(brazil)AND(medicinal plants)AND(prevent OR treatment)

Pubmed n = 46 - ((((((malaria) AND (brazil)) AND (medicinal plants)) AND (prevent)) OR (treatment))) NOT (review)

Embase n = 33 - (malaria AND brazil AND 'medicinal plant' AND prevent OR treatment NOT review)/br

Web of Science n = 50 - (((((ALL=(malaria)) AND ALL=(brazil)) AND ALL=(medicinal plant)) AND ALL=(prevent)) OR ALL=(treatment)) NOT ALL=(review)

BVS/Lilacs n = 50 - (malaria) AND (brazil) AND (medicinal plants) AND (prevent) OR (treatment) AND NOT (review)

Scielo n = 15 - (malaria) AND (brazil) AND (medicinal plants) AND (prevent) OR (treatment) AND NOT (review)

Google Scholar n = 35 - (malaria) AND (brazil) AND (medicinal plants) AND (prevent) OR (treatment) AND NOT (review)

TABLE I

PRISMA checklist

Section and topic	Item #	Checklist item	Location where item is reported
**TITLE**			
Title	1	Identify the report as a systematic review.	1
**ABSTRACT**			
Abstract	2	See the PRISMA 2020 for Abstracts checklist.	3
**INTRODUCTION**			
Rationale	3	Describe the rationale for the review in the context of existing knowledge.	4
Objectives	4	Provide an explicit statement of the objective(s) or question(s) the review addresses.	5
**METHODS**			
Eligibility criteria	5	Specify the inclusion and exclusion criteria for the review and how studies were grouped for the syntheses.	6
Information sources	6	Specify all databases, registers, websites, organisations, reference lists and other sources searched or consulted to identify studies. Specify the date when each source was last searched or consulted.	6
Search strategy	7	Present the full search strategies for all databases, registers and websites, including any filters and limits used.	6
Selection process	8	Specify the methods used to decide whether a study met the inclusion criteria of the review, including how many reviewers screened each record and each report retrieved, whether they worked independently, and if applicable, details of automation tools used in the process.	7
Data collection process	9	Specify the methods used to collect data from reports, including how many reviewers collected data from each report, whether they worked independently, any processes for obtaining or confirming data from study investigators, and if applicable, details of automation tools used in the process.	7
Data items	10a	List and define all outcomes for which data were sought. Specify whether all results that were compatible with each outcome domain in each study were sought (e.g. for all measures, time points, analyses), and if not, the methods used to decide which results to collect.	7

	10b	List and define all other variables for which data were sought (e.g. participant and intervention characteristics, funding sources). Describe any assumptions made about any missing or unclear information.	7
Study risk of bias assessment	11	Specify the methods used to assess risk of bias in the included studies, including details of the tool(s) used, how many reviewers assessed each study and whether they worked independently, and if applicable, details of automation tools used in the process.	7
Effect measures	12	Specify for each outcome the effect measure(s) (e.g. risk ratio, mean difference) used in the synthesis or presentation of results.	7
Synthesis methods	13a	Describe the processes used to decide which studies were eligible for each synthesis (e.g. tabulating the study intervention characteristics and comparing against the planned groups for each synthesis (item #5)).	N/A
	13b	Describe any methods required to prepare the data for presentation or synthesis, such as handling of missing summary statistics, or data conversions.	N/A
	13c	Describe any methods used to tabulate or visually display results of individual studies and syntheses.	N/A
	13d	Describe any methods used to synthesise results and provide a rationale for the choice(s). If meta-analysis was performed, describe the model(s), method(s) to identify the presence and extent of statistical heterogeneity, and software package(s) used.	N/A
	13e	Describe any methods used to explore possible causes of heterogeneity among study results (e.g. subgroup analysis, meta-regression).	N/A
	13f	Describe any sensitivity analyses conducted to assess robustness of the synthesised results.	N/A
Reporting bias assessment	14	Describe any methods used to assess risk of bias due to missing results in a synthesis (arising from reporting biases).	N/A
Certainty assessment	15	Describe any methods used to assess certainty (or confidence) in the body of evidence for an outcome.	N/A
**RESULTS**			
Study selection	16a	Describe the results of the search and selection process, from the number of records identified in the search to the number of studies included in the review, ideally using a flow diagram.	8

	16b	Cite studies that might appear to meet the inclusion criteria, but which were excluded, and explain why they were excluded.	8
Study characteristics	17	Cite each included study and present its characteristics.	8
Risk of bias in studies	18	Present assessments of risk of bias for each included study.	Table S2
Results of individual studies	19	For all outcomes, present, for each study: (a) summary statistics for each group (where appropriate) and (b) an effect estimate and its precision (e.g. confidence/credible interval), ideally using structured tables or plots.	8-14
Results of syntheses	20a	For each synthesis, briefly summarise the characteristics and risk of bias among contributing studies.	8-14
	20b	Present results of all statistical syntheses conducted. If meta-analysis was done, present for each the summary estimate and its precision (e.g. confidence/credible interval) and measures of statistical heterogeneity. If comparing groups, describe the direction of the effect.	8-14
	20c	Present results of all investigations of possible causes of heterogeneity among study results.	8-14
	20d	Present results of all sensitivity analyses conducted to assess the robustness of the synthesised results.	8-14
Reporting biases	21	Present assessments of risk of bias due to missing results (arising from reporting biases) for each synthesis assessed.	Table S2
Certainty of evidence	22	Present assessments of certainty (or confidence) in the body of evidence for each outcome assessed.	8-14
**DISCUSSION**			
Discussion	23a	Provide a general interpretation of the results in the context of other evidence.	14-17
	23b	Discuss any limitations of the evidence included in the review.	14-17
	23c	Discuss any limitations of the review processes used.	14-17
	23d	Discuss implications of the results for practice, policy, and future research.	14-17
**OTHER INFORMATION**			
Registration and protocol	24a	Provide registration information for the review, including register name and registration number, or state that the review was not registered.	5
	24b	Indicate where the review protocol can be accessed, or state that a protocol was not prepared.	5

	24c	Describe and explain any amendments to information provided at registration or in the protocol.	N/A
Support	25	Describe sources of financial or non-financial support for the review, and the role of the funders or sponsors in the review.	18
Competing interests	26	Declare any competing interests of review authors.	18
Availability of data, code and other materials	27	Report which of the following are publicly available and where they can be found: template data collection forms; data extracted from included studies; data used for all analyses; analytic code; any other materials used in the review.	18

Source: Page MJ, McKenzie JE, Bossuyt PM, Boutron I, Hoffmann TC, Mulrow CD, et al. The PRISMA 2020 statement: an updated guideline for reporting systematic reviews. BMJ. 2021; 372: n71. doi: https://doi.org/10.1136/bmj.n71.

TABLE II

The main characteristics of Brazilian plants studied as potential antimalarials

Reference	Popular name of the plant	Species	Plant family	Biome	Quality of studies*
(15)	Caferana	*Picrolemma sprucei*	Simaroubaceae	Amazon Rainforest	7
	Araracanga and Quina-da-mata	*Aspidosperma desmanthum*	Apocynaceae	Amazon Rainforest	
	Amarelão	*Aspidosperma vargasii*	Apocynaceae	-	
	Caapeba, Capeba and Capeba-branca	*Pothomorphe peltata*	Piperaceae	Amazon Rainforest, Cerrado	
(16)	Flor-de-sapo	*Holostylis reniformis*	Aristolochiaceae	Caatinga, Cerrado and Atlantic Forest	7
(17)	Guatambu	*Aspidosperma ramiflorum*	Apocynaceae	Atlantic Forest	7
(18)	N/A	*Homalolepis suffruticosa*	Simaroubaceae	Cerrado	7
(19)	Faveira	*Poincianella pluviosa*	Fabaceae	Cerrado and Pantanal	7
(20)	Peroba-Vermelha	*Aspidosperma olivaceum*	Apocynaceae	Atlantic Forest	8
(21)	Guatambú-do-cerrado and Pereiro-do-campo	*Aspidosperma tomentosum*	Apocynaceae	Cerrado	5
	Jacareúba, Landim, Guanandi and Jacareúba	*Calophyllum brasiliense*	Calophyllaceae	Amazon Rainforest, Caatinga, Cerrado, Atlantic Forest	
	Sangra-d’água, Urucurana and Pau-de-sangue	*Croton urucurana*	Euphorbiaceae	Amazon Rainforest, Cerrado, and Atlantic Forest	
	Caquizeiro-do-cerrado, Marmelada-brava and fruta-de-jacu	*Diospyros hispida*	Ebenaceae	Cerrado and Atlantic Forest	
	Pau-Santo	*Kielmeyera coriacea*	Clusiaceae	Amazon Rainforest and Cerrado	
	Mata-cachorro and Paraíba	*Simarouba versicolor*	Simaroubaceae	Amazon Rainforest, Caatinga and Cerrado	
	Manacá and Manacá-do-cerrado	*Spiranthera odoratissima*	Rutaceae	Amazon Rainforest, Caatinga and Cerrado	
	Aroeira-mansa, Aroeira-vermelha and Aroeira-do-paraná	*Schinus terebinthifolius*	Anacardiaceae	Cerrado, Atlantic Forest and Pampa	
(22)	Copaíba	*Copaifera reticulata*	Fabaceae	Amazon Rainforest	8
(23)	Guatambú and peroba	*Aspidosperma parvifolium*	Apocynaceae	Atlantic Forest	6
(24)	Açaizeiro	*Euterpe oleracea*	Arecaceae	N/A	7
(25)	Faveira	*Caesalpinia pluviosa*	Fabaceae	Cerrado, Pantanal	7
(45)	Flor-de-urubu	*Amasonia campestris*	Lamiaceae	Amazon Rainforest, Caatinga, Cerrado and Atlantic Forest	8

(46)	Fura-capa, Picão-preto, Erva-picão, Picão do campo, Piolho-de-padre, Cuambú, Carrapicho-de-duas-pontas, Macela-do-campo, Goambú and Picão	*Bidens pilosa*	Asteraceae	Cerrado, Atlantic Forest, Pampa and Pantanal	4
(47)	Laranjeira-do-mato, Mamoninha, Três-folhas and Três-folhas-do-mato	*Esenbeckia febrifuga*	Rutaceae	Cerrado and Atlantic Forest	7
(48)	Carapanaúba	*Aspidosperma nitidum*	Apocynaceae	Amazon Rainforest	8
(49)	"pico-pico, picão-preto, fura-capa"	*Cucurbita maxima*	Cucurbitaceae	Caatinga, Cerrado, Atlantic Forest e Pampa	4
	Curcuma	*Momordica charantia*	Cucurbitaceae	Amazon Rainforest, Caatinga, Cerrado, Atlantic Forest and Pantanal	
(50)	Caapeba	*Pothomorphe umbellata*	Piperaceae	Amazon Rainforest, Cerrado, and Atlantic Forest	8
	Caapeba, Capeba and Capeba-branca	*Pothomorphe peltata*	Piperaceae	Amazon Rainforest and Cerrado	
(51)	Fel-da-terra	*Deianira erubescens*	Gentianaceae	Cerrado and Atlantic Forest	7
	N/A	*Remijia ferruginea*	Rubiaceae	Amazon Rainforest, Cerrado and Atlantic Forest	
	Quina	*Strychnos pseudoquina*	Loganiaceae	Cerrado	
(52)	Fura-capa, Picão-preto, Erva-picão, Picão do campo, Piolho-de-padre, Cuambú, Carrapicho-de-duas-pontas, Macela-do-campo, Goambú and Picão	*Bidens pilosa*	Asteraceae	Amazon Rainforest, Caatinga, Cerrado, Atlantic Forest, Pampa and Pantanal	7
(53)	Pimenta-longa	*Piper tuberculatum*	Piperaceae	Amazon Rainforest, Caatinga, Cerrado, Atlantic Forest and Pantanal	6
(54)	Sucupira-preto	*Bowdichia virgilioides*	Fabaceae	Amazon Rainforest, Caatinga, Cerrado, Atlantic Forest and Pantanal	2
(55)	Pau-ferro and Jucá	*Libidibia ferrea*	Fabaceae	Caatinga, Cerrado and Atlantic Forest	7
(56)	Priprioca	*Cyperus articulatus*	Cyperaceae	Amazon Rainforest, Caatinga, and Atlantic Forest	6
(57)	Araça and Araça-da-várzea	*Myrciaria dubia*	Myrtaceae	Amazon Rainforest and Cerrado	7
(58)	Assa-peixe	*Vernonia brasiliana*	Asteraceae	Amazon Rainforest, Caatinga and Cerrado	3
	Casadinha, Cambará-roxo, Chilca, Erva-de-são-miguel	*Eupatorium squalidum*	Asteraceae	Amazon Rainforest, Caatinga, Cerrado and Atlantic Forest	
	Carrapicho	*Acanthospermum australe*	Asteraceae	Amazon Rainforest, Caatinga, Cerrado, Atlantic Forest, Pampa and Pantanal	

	Laranjeira-do-mato, Mamoninha, Três-folhas and Três-folhas-do-mato	*Esenbeckia febrifuga*	Rutaceae	Cerrado and Atlantic Forest	
	-	*Lisianthus speciosus*	Gentianaceae	Cerrado	
	Caferana	*Tachia guianensis*	Gentianaceae	Amazon Rainforest	
(59)	Erva-de-santa-maria	*Chenopodium ambrosioides*	Amaranthaceae	N/A	8
(60)	Moranga	*Cucurbita maxima*	Cucurbitaceae	Amazon Rainforest and Cerrado	5
	Melão-são-caetano	*Momordica charantia*	Cucurbitaceae	Amazon Rainforest, Caatinga, Cerrado, Atlantic Forest and Pantanal	5
	N/A	*Platanus acerifolia*	Platanaceae	N/A	7
	Macieira	*Malus domestica*	Rosaceae	N/A	7
(61)	Acanga, Araticum, Araticum-do-mata and Tapanahuacanga	*Annona crassiflora*	Annonaceae	Amazon Rainforest, Cerrado and Pantanal	6
	Alathê, Orelha-de-burro and Pinha-do-campo	*Duguetia furfuracea*	Annonaceae	Amazon Rainforest, Caatinga, Cerrado and Atlantic Forest	
	Envira-chichi, Envira-folha-fina, Pindaíba and Pindaíba-preta	*Xylopia emarginata*	Annonaceae	Amazon Rainforest, Cerrado and Atlantic Forest	
	Bananinha, Begerecum, Cedro-do-campo, Pimenta-de-macaco and Pindaíba-do-campo	*Xylopia aromatica*	Annonaceae	Amazon Rainforest, Cerrado	
	Guaçatonga and Pau-de-lagarto	*Casearia sylvestris*	Flacourtiaceae	Amazon Rainforest, Caatinga, Cerrado, Atlantic Forest, Pampa, Pantanal	
	Arco-de-barril and Rabo-de-bugio	*Cupania vernalis*	Sapindaceae	Amazon Rainforest, Caatinga, Cerrado, Atlantic Forest, Pampa and Pantanal	
	Brazeiro, Camboatã, Olho-de-cotia, Batabaiba and Pau-da-digestão	Matayba guianensis	Sapindaceae	Amazon Rainforest, Cerrado, Atlantic Forest and Pantanal	
	Pereiro	*Aspidosperma macrocarpon*	Apocynaceae	Cerrado	
(62)	Peroba-poca, Peroba-iquira, Peroba-de-lagoa-santa, Peroba-de-minas and Peroba-rosa	*Aspidosperma cylindrocarpon*	Apocynaceae	Amazon Rainforest, Cerrado, and Atlantic Forest	7
	Guatambu	*Aspidosperma parvifolium*	Apocynaceae	Atlantic Forest	
	Peroba-vermelha	*Aspidosperma olivaceum*	Apocynaceae	Atlantic Forest	
	Guatambu	*Aspidosperma ramiflorum*	Apocynaceae	Atlantic Forest	
	Amargoso and Araracanga	*Aspidosperma spruceanum*	Apocynaceae	Amazon Rainforest	
	Peroba and Perobinha	*Aspidosperma tomentosum*	Apocynaceae	Cerrado	

(63)	Guatambú and peroba	*Aspidosperma parvifolium*	Apocynaceae	Atlantic Forest	6
(64)	Acajaiba and Cajueiro	*Anacardium occidentale*	Anacardiaceae	Amazon Rainforest, Caatinga, Cerrado, Atlantic Forest, Pampa and Pantanal	7
	Capim-membeca and Capim-colchão	*Andropogon leucostachyus*	Poaceae	Amazon Rainforest, Caatinga, Cerrado, Atlantic Forest, Pampa and Pantanal	
	-	*Clidemia bullosa*	Melastomataceae	Amazon Rainforest, Cerrado	
	Marassacaca and Sacaca	*Croton cajucara*	Euphorbiaceae	Amazon Rainforest	
	Timuatã	*Derris floribunda*	Fabaceae	Amazon Rainforest and Cerrado	
	Pixirica	*Miconia nervosa*	Melastomataceae	Amazon Rainforest, Caatinga, Cerrado and Atlantic Forest	
	Anjico, Fava-pé-de-arara, Faveira-benguê and Fava-pé-de-arara	*Parkia nítida*	Fabaceae	Amazon Rainforest, Caatinga, Cerrado, and Atlantic Forest	
	Guaraná	*Paullinia cupana*	Sapindaceae	Amazon Rainforest	
	Cipó-cururu	*Stigmaphyllon sinuatum*	Malpighiaceae	Amazon Rainforest	
	N/A	*Xylopia amazonica*	Annonaceae	Amazon Rainforest	
	N/A	*Zanthoxylum djalma-batistae*	Rutaceae	Amazon Rainforest	
(65)	Andiroba	*Carapa guianensis*	Meliaceae	Amazon Rainforest	8
(66)	N/A	*Aspidosperma sp.*	Apocynaceae	Amazon Rainforest, Caatinga, Cerrado, Atlantic Forest, Pampa and Pantanal	7
(67)	Candeeiro	*Vanillosmopsis arborea*	Asteraceae	Caatinga and Cerrado	5
	Alecrim-pimenta	*Lippia sidoides*	Verbanaceae	Amazon Rainforest, Caatinga, Cerrado, Atlantic Forest and Pantanal	
	Alecrim-de cabocla and Canelinha	*Croton zehntneri*	Euphorbiaceae	Caatinga	
(68)	Carapanaúba	*Aspidosperma excelsum*	Apocynaceae	Amazon Rainforest	8
(69)	Fura-capa, Picão-preto, Erva-picão, Picão do campo, Piolho-de-padre, Cuambú, Carrapicho-de-duas-pontas, Macela-do-campo, Goambú and Picão	*Bidens pilosa*	Asteraceae	Amazon Rainforest, Caatinga, Cerrado, Atlantic Forest, Pampa and Pantanal	8
(70)	Cedro	*Simaba cedron*	Simaroubaceae	Amazon Rainforest and Atlantic Forest	2
	Carapanaúba-amarela and Carapanaúba-do-baixio	*Aspidosperma rigidum*	Apocynaceae	Amazon Rainforest	
	Castanheira, Castanha-do brasil and Castanha-do-pará	*Bertholletia excelsa*	Lecitidacea	Amazon Rainforest	

	Aturiá and Juquiri-preto	*Machaerium ferox*	Fabaceae	Amazon Rainforest	
	Cervejeira and Saracurá-mirá	*Ampelozizyphus amazonicus*	Rhamnaceae	Amazon Rainforest	
	Mangueira	*Mangifera indica*	Anacardiaceae	Amazon Rainforest, Atlantic Forest, Cerrado, Caatinga, Pampa and Pantanal	
	Batatão	*Operculina hamiltonii*	Convolvulaceae	Amazon Rainforest, Caatinga, Cerrado, Atlantic Forest and Pantanal	
	Andiroba	*Carapa guianensis*	Meliaceae	Amazon Rainforest	
	Unha-de-gato and Esperaí	*Uncaria guianensis*	Rubiaceae	Amazon Rainforest and Cerrado	
	Açaizeiro	*Euterpe oleracea*	Arecaceae	Amazon Rainforest and Cerrado	
	Uxi	*Endopleura uchi*	Humiriaceae	Amazon Rainforest	
(71)	Andiroba	*Carapa guianensis*	Meliaceae	Amazon Rainforest	6
(72)	Acanga, Araticum-do-mata and Tapanahuacanga	*Annona crassiflora*	Annonaceae	Amazon Rainforest, Cerrado and Pantanal	7
(73)	Caapeba, Capeba and Capeba-branca	*Pothomorphe peltata*	Piperaceae	Amazon Rainforest and Cerrado	7
(74)	Caapeba, Capeba and Capeba-branca	*Piper peltatum*	Piperaceae	Amazon Rainforest and Cerrado	3
(75)	Camapú, Balão-rajado, Balão-rajado and Joá-de-capote	*Physalis angulata*	Solanaceae	Amazon Rainforest, Caatinga, Cerrado, Atlantic Forest, Pampa and Pantanal	8
(76)	Para-tudo, Quina and Quina-do-campo	*Hortia oreadica*	Rutaceae	Amazon Rainforest and Cerrado	5
(77)	-	*Tachia sp.*	Gentianaceae	Amazon Rainforest	8
(78)	Priprioca	*Cyperus articulatus*	Cyperaceae	Amazon Rainforest, Caatinga, and Atlantic Forest	7
(79)	Pimenta-longa	*Piper tuberculatum*	Piperaceae	Amazon Rainforest, Caatinga, Cerrado, Atlantic Forest and Pantanal	6
	Pimenta-do -Reino	*Piper nigrum*	Piperaceae	Amazon Rainforest, Caatinga, Cerrado, Atlantic Forest, Pampa and Pantanal	6
(80)	Pitiá	*Aspidosperma ulei*	Apocynaceae	Amazon Rainforest	7
(81)	Embaúba-vermelha	*Cecropia glaziovii*	Urticaceae	Atlantic Forest	7
(82)	Melão-são-caetano	*Momordica charantia*	Cucurbitaceae	Amazon Rainforest, Caatinga, Cerrado, Atlantic Forest and Pantanal	3
(83)	Sucuuba and Janaguba	*Himatanthus articulatus*	Apocynaceae	Amazon Rainforest and Cerrado	5
(84)	Marupá and Tiriricão	*Eleutherine plicata*	Iridaceae	Amazon Rainforest, Cerrado, and Atlantic Forest	7
(85)	Vassoura	*Symphyopappus casarettoi*	Asteraceae	Atlantic Forest	8

N/A: non-applicable. *The studies were evaluated using a scoring system that ranges from 0 to 3 for low quality, 4 to 6 for moderate quality, and 7 to 8 for high quality.[13]

TABLE III

Phytochemistry aspects of the plant families, compounds, and parts of the plants found in the selected studies

Reference	Plant family	Active compounds	Parts of the plant used in the studies
(15)	Simaroubaceae<br>Apocynaceae<br>Piperaceae	Simaroubaceae - quassinoids (neosergeolide)<br>Apocynaceae - alkaloids (aspidocarpine)<br>Piperaceae: 4-Nerolidylcatechol	Simaroubaceae: Roots and stems<br>Apocynaceae: Bark<br>Piperaceae: Roots
(16)	Aristolochiaceae	Phenolics (polyphenols)	Roots, stems, and leaves
(17)	Apocynaceae	Alkaloids (isositsirikine)	Stem bark and leaves
(18)	Simaroubaceae	Alkaloids (canthinone); squalene and potolimonoid-type triterpenes	Roots
(19)	Fabaceae	Phenolics (ellagic acid)	Leaves
(20)	Apocynaceae	Alkaloids (aspidoscarpine, uleine, apparicine, and N-methyl-tetrahydrolivacine)	Bark and leaves
(21)	Apocynaceae<br>Calophyllaceae<br>Euphorbiaceae<br>Ebenaceae<br>Clusiaceae<br>Simaroubaceae<br>Rutaceae<br>Anacardiaceae	N/A	Apocynaceae: Roots<br>Calophyllaceae: Root wood and root bark<br>Euphorbiaceae: Stem wood<br>Ebenaceae: Root and stem bark<br>Clusiaceae: Stem bark<br>Anacardiaceae, Simaroubaceae: Leaves<br>Rutaceae: Leaves and roots
(22)	Fabaceae	Sesquiterpenes and diterpenes (β-caryophyllene, β-bisabolene)	Oleoresin
(23)	Apocynaceae	Monoterpenes	Bark and stem
(24)	Arecaceae	Phenolics (Cyanidin-3-glucoside, cyanidin-3-rutinoside, protocatechuic acid, orientin, isovitexin, scoparin, and isoorientin)	Pulp
(25)	Fabaceae	Phenolics (ellagic acid), saponins (sapponone B, protosappanin C), flavonoids (quercetin), 3-deoxy-4-O-methylepisappanol, voucapen-5a-ol	Bark
(45)	Lamiaceae	Alkaloids, phenols, and tannins, anthocyanin heterosids	Roots
(46)	Asteraceae	Phenolics (phenylacetylene)	Whole plant, stems, leaves and roots
(47)	Rutaceae	Furoquinolines and acridone alkaloids, limonoids, and coumarins	Stem

(48)	Apocynaceae	Alkaloids	Wood bark, branches, and leaves
(49)	Cucurbitaceae	N/A	Seeds and leaves
(51)	Gentianaceae<br>Rubiaceae<br>Loganiaceae	Gentianaceae: flavonoids and phenolics (tannins)<br>Rubiaceae: alkaloids<br>Loganiaceae: alkaloids	Gentianaceae: Roots and leaves<br>Loganiaceae, Rubiaceae: Bark
(52)	Asteraceae	Flavonoids and polyacetylenes	Roots
(53)	Piperaceae	Alkaloids (piplartine) (E)-1-morpholin-4-yl-3-(3,4-dimethoxyphenyl)prop-2-en-1-one; (E)-1-morpholin-4-yl-3-(3,4,5-trimethoxyphenyl)prop-2-en-1-one; (E)-3-(3,4-dimethoxyphenyl)-N-pentylprop-2-enamide; (E)-3-(3,4,5-trimethoxyphenyl)-N-pentylprop-2-enamide.	Roots
(54)	Fabaceae	N/A	Bark
(55)	Fabaceae	Phenolics (gallic acid, gallotannin, valoneic acid dilactone, ellagic acid)	Pods
(56)	Cyperaceae	Sesquiterpenes (corymbolone, cyclocolorenone and cadalene)	Rhizome
(57)	Myrtaceae	Phenolics (tannins and anthocyanins)	Seeds
(58)	Asteraceae<br>Rutaceae<br>Gentianaceae	N/A	Asteraceae: Aerial plant, whole plant and leaves<br>Rutaceae: Stalk bark<br>Gentianaceae: Roots
(59)	Amaranthaceae	N/A	Leaves
(60)	Platanaceae<br>Rosaceae	Plantanaceae: triterpenes (betulinic acid, 3β-hydroxy-lup-20 (29)-en-28-oic acid)<br>Rosaceae: triterpenes (ursolic acid, 3β-hydroxyurs-12-en-28-oic acid analogues)	Platanaceae: Bark<br>Rosaceae: Fruit peels
(61)	Annonaceae<br>Flacourtiaceae<br>Sapindaceae<br>Apocynaceae	N/A	Annonaceae: Root bark, root stem, and root wood<br>Flacourtiaceae: Stem wood<br>Sapindaceae: Root bark and leaves<br>Apocynaceae: Root bark
(62)	Apocynaceae	Alkaloids	Trunk, leaves, fruits, bark and seeds
(63)	Apocynaceae	Monoterpenes	Bark
(64)	Anacardiaceae<br>Poaceae<br>Melastomataceae	N/A	Anacardiaceae, Euphorbiaceae, Fabaceae, Melastomataceae, Sapindaceae: Bark, leaves<br>Poaceae: Aerial plant

	Euphorbiaceae<br>Fabaceae<br>Melastomataceae<br>Fabaceae<br>Sapindaceae<br>Malpighiaceae<br>Annonaceae<br>Rutaceae		Annonaceae, Melastomataceae, Rutaceae: Leaves, branch<br>Malpighiaceae: leaves
(65)	Meliaceae	Limonoids	Seeds
(66)	Apocynaceae	Alkaloids	N/A
(67)	Asteraceae<br>Verbenaceae<br>Euphorbiaceae	Monoterpenes and sesquiterpenes	Asteraceae: Stem<br>Verbenaceae, Euphorbiaceae: Leaves
(68)	Apocynaceae	Alkaloids	Trunk bark
(69)	Asteraceae	Flavonoids and polyacetylenes	Roots
(70)	Simaroubaceae<br>Apocynaceae<br>Lecitidacea<br>Fabaceae<br>Rhamnaceae<br>Anacardiaceae<br>Convolvulaceae<br>Meliaceae<br>Rubiaceae<br>Arecaceae<br>Humiriaceae	N/A	Apocynaceae, Anacardiaceae, Humiriaceae, Rhamnaceae, Rubiaceae, Simaroubaceae: Bark<br>Lecitidacea: Fruit peel, bark and bast<br>Fabaceae: Stalk<br>Convolvulaceae: Potato<br>Meliaceae: Seeds<br>Arecaceae: Roots
(71)	Meliaceae	Limonoids (6α-acetoxyazadiradione (1), andirobin (2), 6α-acetoxygedunin (3), 7-deacetyl-7-oxogedunin (4), 6α-hydroxydeacetylgedunin (5), chloroquine diphosphate, aquinine sulfate)	Seeds

(72)	Annonaceae	Alkaloids, flavonoids, acetogenins	Wood
(73)	Piperaceae	4-Nerolidylcatechol and semi-synthetic derivatives	Roots
(74)	Piperaceae	4-Nerolidylcatechol (4-NC)	Roots
(75)	Solanaceae	Physalins B (1), D (2), F (3), and G (4)	N/A
(76)	Rutaceae	Methyl 5,7-dimethoxy-2,2-dimethyl2H -1-benzopyran-6-propanoate	Leaves
(77)	Gentianaceae	Monoterpenes (amplexine)	Roots
(78)	Cyperaceae	Sesquiterpenes (mustakone)	Rhizome
(79)	Piperaceae	Alkaloids (piplartine, piperine)	Roots, Fruits
(80)	Apocynaceae	Alkaloids	Leaves, bark, trunk wood, root wood, and root bark
(81)	Urticaceae	Triterpenes (tormentic acid)	Wood, leaves, and roots
(82)	Cucurbitaceae	N/A	Leaves and stem
(83)	Apocynaceae	Iridoids plumieride	Bark
(84)	Iridaceae	Naphtohoquinones (eleutherin and isoleutherin)	Bulb
(85)	Asteraceae	Flavonoids (BP-181-7, BP204 (caryatin))	Inflorescences

N/A: non-applicable.

TABLE IV

In vitro aspects of the active compounds of the studied Brazilian plants

Reference	*Plasmodium* strain	Tested compound	% parasitaemia reduction	IC50 (Mean±SD)	Selectivity Index (SI) (cell line*)
(15)	*P. falciparum* K1	Neosergeolide<br>Ellipticine<br>Aspidocarpine<br>4-Nerolidylcatechol	N/A	0.001 μg/mL<br>0.018 μg/mL<br>0.007 μg/mL<br>0.21 μg/mL	N/A
(16)	*P. falciparum* BHz26/86	(7’R,8S,8’R)-3’,4,4’,5-Tetramethoxy-2,7’-cyclolignan-7-one<br>(7’R,8R,8’S)-3’,4,4’,5-Tetramethoxy-2,7’-cyclolignan-7-one<br>(7’R,8S,8’S)-3’,4,4’,5-Tetramethoxy-2,7’-cyclolignan-7-one<br>(7’R,8S,8’S)-3’,4’-Dimethoxy-4,5-methylenodioxy-2,7’-cyclolignan-7-one<br>(7’R,8S,8’S)-4,5-Dimethoxy-3’,4’-methylenodioxy-2,7’-cyclolignan-7-one<br>(7’R,8S,8’R)-4,5-Dimethoxy-3’,4’-methylenodioxy-2,7’-cyclolignan-7-one	N/A	0.32 ± 0.11 μg/mL<br>0.20 ± 0.09 μg/mL<br>0.63 ± 0.20 μg/mL<br>8.00 ± 0.65 μg/mL<br>> 140.00 μg/mL<br>0.26 ± 0.08 μg/mL	N/A

(17)	*P. falciparum* W2	Acid fraction (bark)	N/A	2.5C 1.2 µg/mL	138 (BGM)<br>41 (HepG2)<br>135 (PBMC)
		Neutral fraction (bark)	N/A	0.7 ± 0.5 µg/mL	34 (BGM)<br>39 (HepG2)
		Basic fraction (bark)	N/A	0.8 ± 0.5 µg/mL	64 (BGM)<br>62 (HepG2)<br>22 (PBMC)
		Neutral precipitate (bark)	N/A	0.7 ± 0.4 µg/mL	43 (BGM)<br>64 (HepG2)<br>19 (PBMC)
		Methanolic residue (bark)	N/A	1.5 ± 0.9 µg/mL	52 (BGM)<br>47 (HepG2)<br>15 (PBMC)
		Nonsoluble (bark)	N/A	3.1 ± 1.8 µg/mL	200 (BGM)<br>56 (HepG2)<br>48 (PBMC)
		Isositsirikine	N/A	0.2 ± 0.0 µg/mL	83 (BGM)<br>113 (HepG2)
		10-MG	N/A	0.4 ± 0.3 µg/mL	Toxic (HepG2)
		Ramiflorine B	N/A	0.9 ± 0.9 µg/mL	Toxic (BGM)<br>Toxic (HepG2)
		Methanol extract (leaf)	N/A	1.4 ± 0.7 µg/mL	27 (BGM)<br>38 (HepG2)<br>18 (PBMC)
		Acetone extract (leaf)	N/A	1.4 ± 0.4 µg/mL	22 (BGM)<br>39 (HepG2)
(18)	*P. falciparum* W2	Milemaronol<br>meso-Teurilene<br>Hispidol A plus Hispidol B<br>α-Dihydronylocytine plus β-dihydronylocytine<br>5-methoxycanthin-6-one<br>Methanol extract	N/A	10.80 ± 1.49 µg/mL<br>7.89 ± 1.95 µg/mL<br>24.58 ± 1.79 µg/mL<br>26.65 ± 2.40 µg/mL<br>0.0548 ± 0.008 µg/mL<br>1.88 ± 0.56 µg/mL	4.75<br>7.15<br>1.09<br>0.56<br>11.31<br>22.30

(19)	*P. falciparum* W2	Ethanol extract	N/A	6.88 ± 1.64 µg/mL	>145,3 (HepG2)
		Acetate fraction		1.83 µg/mL	>546,4 (HepG2)
		Acqueous fraction		>50 µg/mL	N/A
		Gallic acid		0.215 ± 0.007 µg/mL	>20-40 (HepG2)
		Valoneic acid dilactone		25 – 50 µg/mL	>2.0-4.1 (HepG2)
		Ellagic acid		25 – 50 µg/mL	>4651.1 (HepG2); 414.7 (MPM)
(20)	*P. falciparum* W2	Methanol extract (bark)	N/A	10.6 ± 3.0 µg/mL	17 (HepG2)
		Acid fraction (bark)		6.4 ± 2.3 µg/mL	52 (HepG2)
		Neutral fraction (bark)		2.0 ± 0.2 µg/mL	10 (HepG2)
		Basic fraction (bark)		4.3 ± 2.5 µg/mL	<6 (HepG2)
		Neutral precipitate (bark)		6.7 ± 3.3 µg/mL	68 (HepG2)
		Methanol extract (leaf)		7.2 ± 2.3 µg/mL	126 (HepG2)
		Basic residue (leaf)		4.5 ± 0.7 µg/mL	92 (HepG2)
		Acid fraction (leaf)		8.5 ± 3.5 µg/mL	52 (HepG2)
		Aspidocarpine		5.4 ± 2.5 µg/mL	56 (HepG2)
		Uleine		7.0 ± 0.0 µg/mL	18 (HepG2)
		Apparicine		3.0 ± 1.4 µg/mL	14 (HepG2)
		N-methyl-tetrahydrolivacine		5.7 ± 3.3 µg/mL	22 (HepG2)
(21)	*P. falciparum* FcB1	Dichloromethane extract (*C. brasiliense* root bark)	N/A	9.5 ± 0.02 µg/mL	14.4
		Hexane extract (*C. urucurana* stem wood)		3.5 ± 0.02 µg/mL	>285.7
		Ethyl acetate extract (*D. hispida* roots)		1 ± 0.9 µg/mL	435.8
		Dichloromethane extract (*D. hispida* stem bark)		9.6 ± 3.4 µg/mL	>104.1
		Dichloromethane extract (*K. coriaceae* stem bark)		8.7 ± 0.66 µg/mL	32.8
		Dichloromethane extract (*S. versicolor* leaves)		3.1 ± 0.01 µg/mL	64.4
		Ethyl acetate extract (*S. odoratissima* leaves)		9.2 ± 1.8 µg/mL	20.3
		Hexane extract (*S. odoratissima* roots)		5.5 ± 1.6 µg/mL	34.8
		Dichloromethane extract (*S. terebinthifolius* leaves)		6.4 ± 0.86 µg/mL	32
		Dichloromethane extract (*A. tomentosum* roots)		6.7 ± 0.5 µg/mL	67.5
		Dichloromethane extract (*C. brasiliense* root wood)		6.7 ± 0.87 µg/mL	121.4
(22)	*P. falciparum* W2 and 3D7	Oleoresin	N/A	1.66 ± 0.04 µg/ml (W2)	>60 (WI26VA-4)
				2.54 ± 0.05 µg/ml (3D7)	>39 (WI26VA-4)

(23)	*P. falciparum* W2	Ethanol extract (bark)	N/A	3 ± 3 μg/mL	≥333 (BGM)<br>137 (HepG2)<br>≥333 (PBMC)
		Ethyl acetate fraction (bark)	N/A	9 ± 3 μg/mL	≥111 (BGM)<br>54 (HepG2)<br>48 (PBMC)
		Ethyl acetate fraction (stem)	N/A	6 ± 1 μg/mL	≥167 (BGM)<br>53 (HepG2)<br>26 (PBMC)
		Chloroform fraction (stem)	N/A	5 ± 3 μg/mL	≥200 (BGM)<br>84 (HepG2)<br>29 (PBMC)
(24)	*P. falciparum* HB3 and Dd2	Total phenolics<br><br>Nonanthocyanin phenolics<br>(10 mg/L)<br>(10 mg/L)<br>(20 mg/L)<br>(20 mg/L)<br><br>Total anthocyanins	N/A<br><br><br>24,4% (HB3)<br>34,9% (Dd2)<br>34,2% (HB3)<br>38,3% (Dd2)<br><br>N/A	N/A	N/A

(25)	*P. falciparum* 3D7 and S20	Ethanol extract	N/A	4.84 ± 0.17 µg/mL (3D7<br>3.41 ± 2.45 µg/mL (S20)	N/A<br>N/A
		Aqueous fraction	N/A	10.98 ± 6.01 µg/mL (3D7)<br>13.29 ± 2.70µg/mL (S20)	N/A<br>N/A
		Ethyl-acetate fraction	N/A	2.13 ± 0.94 µg/mL (3D7)<br>2.07 ± 1.38µg/mL(S20)	N/A<br>N/A
		50% ethanolic fraction	N/A	4.55 ± 2.05 µg/mL (3D7)<br>5.49 ± 1.26µg/mL (S20)	N/A<br>N/A
		100% ethanolic fraction	N/A	0.72 ± 0.29 µg/mL (3D7)<br>1.25 ± 0.38 µg/mL (S20)	N/A<br>N/A
		50% methanolic fraction	N/A	0.59 ± 0.33 µg/mL (3D7)<br>1.72 ± 0.27 µg/mL (S20)	N/A<br>N/A
		100% methanolic fraction	N/A	1.30 ± 0.43 µg/mL (3D7)<br>3.61 ± 2.46 µg/mL (S20)	N/A<br>N/A
		70% acetone-water fraction	N/A	17.19 µg/mL (3D7)<br>Not determined (S20)	N/A<br>N/A
(45)	*P. falciparum* K1	Ethanol extract:	N/A	42.94 µg/mL	N/A
(46)	P. falciparum BHz 26/86	Ether extract<br>Ethanol extract<br>Chloroform fraction (stem)<br>Chloroform fraction (leaves)<br>Chloroform fraction (roots)<br>Butanol fraction (leaves)<br>Butanol fraction (roots)	33%<br>90%<br>47%<br>94%<br>86%<br>79%<br>68%	N/A	N/A
(47)	*P. falciparum* W2 and 3D7	Flindersiamine	N/A	265.6 ± 12.8 µM (3D7)<br>348.0 ± 35.3 µM (W2)	N/A
		Skimmiamine	N/A	166.0 ± 5.4 µMl (3D7)<br>75.3 ± 2.7 µM (W2)	
		g-Fagarine	N/A	109.8 ± 18.3 µM (3D7)<br>157.2 ± 12.2 µM (W2)	

(48)	*P. falciparum* W2	Ethanol extract (bark)	N/A	7 ± 3 µg/mL[#]<br>4.6 ± 4.4 µg/mL[##]	286 (BGM]); 83 (HepG2) 435 (BGM); 126 (HepG2)
		Methanol extract A (bark)	N/A	8.3 ± 1.6 µg/mL[#]<br>10.7 ± 1.3 µg/mL[##]	116 (BGM); 31 (HepG2)<br>90 (BGM); 24 (HepG2)
		Methanol extract B (bark)	N/A	10 ± 3.6 µg/mL[#]<br>6.6 ± 2.2 µg/mL[##]	200 (BGM); 100 (HepG2)<br>303 (BGM); 126 (HepG2)
		Methanol extract C (bark)	N/A	12.4 ± 1.9 µg/mL[#]<br>10.8 ± 0.4 µg/mL[##]	34 (BGM); 48 (HepG2)<br>40 (BGM); 56 (HepG2)
		Fraction FO III	N/A	1.6 ± 0.06 µg/mL[#]<br>2.3 ± 1.5 µg/mL[##]	188 (BGM); 39 (HepG2)<br>70 (BGM); 30 (HepG2)
		Fraction FO IV	N/A	3.3 ± 0.4 µg/mL[#]<br>4.8 ± 2.1 µg/mL[##]	30 (BGM); 51 (HepG2)<br>19 (BGM); 35 (HepG2)
		Precipitate	N/A	5.5 ± 0.2 µg/mL[#]<br>7.6 ± 4.4 µg/mL[##]	364 (BGM); 201 (HepG2)<br>263 (BGM); 145 (HepG2)
		Ethanol extract (leaves)	N/A	22 ± 0.7 µg/mL[#]<br>25.3 ± 6.0 µg/mL[##]	91 (BGM); 29 (HepG2)<br>79 (BGM); 25 (HepG2)
(52)	*P. falciparum* W2, D6 and BHz	Ethanol extract	N/A	12.6 ± 0.01 µg/mL (W2)<br>10.4 ± 0.01 µg/mL (D6)<br>17.0 ± 0.03 µg/mL (BHz)	N/A
(53)	*P. falciparum* Unb169	Piplartine<br>Cinnamide 2<br>Cinnamide 3<br>Cinnamide 4<br>Cinnamide 5	N/A	3.2 µg/mL<br>39.0 µg/mL<br>173.0 µg/mL<br>41.4 µg/mL<br>6.6 µg/mL	72.5<br>6.8<br>1.5<br>10.7<br>38.4
(54)	*P. falciparum* W2 and 3D7	Aqueous extract	N/A	>50 µg/ml	N/A
(55)	*P. falciparum* W2	Hydroalcoholic extract	N/A	11.10 ± 1.13 µg/mL	>9
(56)	*P. falciparum* W2 and 3D7	Ethanol Extract	N/A	1.21 ± 0.01 µg/mL (W2)<br>1.10 ± 0.06 µg/mL (3D7)	>83<br>>91
(57)	*P. falciparum* W2 and 3D7	100% water extract<br>100% ethanol extract<br>50% ethanol extract<br>75% ethanol extract<br>25% ethanol extract	N/A	37.2 µg/mL (W2); 18.7 µg/mL (3D7)<br>9.5 µg/mL (W2); 8.2 µg/mL (3D7)<br>20.7 µg/mL (W2); 19.8 µg/mL (3D7)<br>12.6 µg/mL (W2); 14 µg/mL (3D7)<br>18.8 µg/mL (W2); 18.7 µg/mL (3D7)	N/A
(58)	*P. falciparum*	N/A	71%	N/A	N/A
(59)	*P. falciparum* W2 and 3D7	Ethanol extract	N/A	25.4 µg/mL	N/A

(60)	*P. falciparum*	Betulinic acid<br>Ursolic acid<br>Betulinic acid 1e semisynthetic<br>Betulinic acid 1f semisynthetic<br>Ursolic acid 2e semisynthetic	N/A	18 ± 0.17 μM<br>36 ± 0.25 μM<br>5 ± 0.14 μM<br>8 ± 0.16 μM<br>7 ± 0.15 μM	N/A<br>N/A<br>>20 (HEK-293)<br>>12.5 (HEK-293)<br>>14.28 (HEK-293)
(61)	*P. falciparum* FcB1	Ethanol extract (D. furfuracea root wood)<br>Hexane extract (X. aromatica stem bark)<br>Hexane extract (X. ermaginata root bark)<br>Hexane extract (X. ermaginata stem bark)<br>Ethanol extract (A. macrocarpon root bark)<br>Hexane extract (C. silvestris var. lingua stem wood)<br>Hexane extract (C. vernalis leaves)<br>Hexane extract (M. guianensis root bark)<br>Hexane extract (A. crassiflora root bark)	N/A	6.2 ± 0.1 μg/mL<br>4.7 ± 0.9 μg/mL<br>4.9 ± 0.2 μg/mL<br>5.2 ± 0.4 μg/mL<br>4.9 ± 1.1 μg/mL<br>0.9 ± 0.2 μg/mL<br>0.9 ± 0.3 μg/mL<br>6.1 ± 0.6 μg/mL<br>7.6 ± 2.1 μg/mL	10.05 (L6); 11.0 (MRC-6)<br>2.0 (L6); 4.2 (MRC-6)<br>4.5 (L6); 6.0 (MRC-6)<br>10.3 (L6); 11.2 (MRC-6)<br>13.9 (L6); 16.2 (MRC-6)<br>1.85 (L6); 1.73 (MRC-6)<br>1.83 (L6); 2.4 (MRC-6)<br>10.0 (L6); >16.4 (MRC-6)<br>5.94 (L6); 4.6 (MRC-6)

(62)	*P. falciparum* W2 and 3D7	Ethanol extract (percolation) (*A. cylindrocarpon* leaves)	N/A	44.0 ± 6.36 µg/ml (W2)<br>39.0 ± 2.83 µg/ml (3D7)	>11.36 (Vero)<br>>12.82 (Vero)
		Dichloromethane fraction (soxhlet) (*A. olivaceum* leaves)	N/A	7.0 ± 0,2 µg/ml (W2)<br>25.5 ± 2.12 µg/ml (3D7)	>71.43 (Vero)<br>>19.61 (Vero)
		Ethanol extract (soxhlet) (*A. olivaceum* leaves)	N/A	7.0 ± 0.71 µg/ml (W2)<br>5.0 ± 2.80 µg/ml (3D7)	N/A<br>N/A
		Dichloromethane fraction (soxhlet) (*A. olivaceum* trunk)	N/A	<6 µg/ml (W2)<br><6 µg/ml (3D7)	>83.33 (Vero)<br>>83.33 (Vero)
		Dichloromethane fraction (soxhlet) (*A. olivaceum* bark)	N/A	<6 µg/ml (W2)<br><6 µg/ml (3D7)	N/A<br>N/A
		Ethanol extract (soxhlet) *(A. olivaceum* bark)	N/A	5.0 ± 2.8 µg/ml (W2)<br>7.0 ± 0.42 µg/ml (3D7)	>100,00 (Vero)<br>>71.43 (Vero)
		Ethanol extract (percolation) (*A. parviflorum* leaves)	N/A	32.75 ± 1.06 µg/ml (W2)<br>20.51 ± 0.70 µg/ml (3D7)	>15.27 (Vero)<br>>24.38 (Vero)
		Ethanol extract (percolation) (*A. ramiflorum* leaves)	N/A	32.8 ± 1.13 µg/ml (W2)<br>20.5 ± 0.71 µg/ml (3D7)	N/A<br>N/A
		Dichloromethane fraction (soxhlet) (*A. ramiflorum* leaves)	N/A	<6 µg/ml (W2)<br><6 µg/ml (3D7)	N/A<br>N/A
		Ethanol extract (percolation) (*A. ramiflorum* trunk)	N/A	36.5 ± 0.20 µg/ml (W2)<br>48.0 ± 1.1 µg/ml (3D7)	N/A<br>N/A
		Dichloromethane fraction (soxhlet) (*A. ramiflorum* trunk)	N/A	N/A (W2)<br>9.5 ± 1.41 µg/ml (3D7)	N/A<br>>52.63 (Vero)
		Ethanol extract (soxhelt) (*A. ramiflorum* trunk)	N/A	19.75 ± 0.35 µg/ml (W2)<br>0.98 ± 0.03 µg/ml (3D7)	N/A<br>N/A
		Dichloromethane fraction (soxhlet) (*A. ramiflorum* bark)	N/A	<6 µg/ml (W2)<br><6 µg/ml (3D7)	>83,33 (Vero)<br>>83.33 (Vero)
		Ethanol extract (percolation) (*A. spruceanum* leaves)	N/A	65.0 ± 4.2 µg/ml (W2)<br>>100 µg/ml (3D7)	18.27 (Vero)<br>>18.27 (Vero)
		Dichloromethane fraction (soxhlet) (*A. spruceanum* leaves)	N/A	23.25 ± 0.35 µg/ml (W2)<br>35.0 ± 4.2 µg/ml (3D7)	N/A<br>N/A

		Ethanol extract (percolation) (*A. spruceanum* trunk)	N/A	29.52 ± 0.71 µg/ml (W2)<br>41.5 ± 2.12 µg/ml (3D7)	N/A<br>N/A
		Dichloromethane fraction (soxhlet) (*A. spruceanum* trunk)	N/A	<6 µg/ml (W2)<br><6 µg/ml (3D7)	N/A<br>N/A
		Dichloromethane fraction (percolation) (*A. spruceanum* trunk)	N/A	37.0 ±7.1 µg/ml (W2)<br>>100 µg/ml (3D7)	N/A<br>N/A
		Ethanol fraction (percolation) (*A. spruceanum* bark)	N/A	26.25 ± 4.07 µg/ml (W2)<br>14.0 ± 4.2 µg/ml (3D7)	N/A<br>N/A
		Dichloromethane fraction (soxhlet) (*A. spruceanum* bark)	N/A	<6 µg/ml (W2)<br>15.75 ± 1.76 µg/ml (3D7)	N/A<br>N/A
		Ethanol fraction (soxhlet) (*A. spruceanum* bark)	N/A	28.01 ± 3.51 µg/ml (W2)<br>19.0 ± 2.83 µg/ml (3D7)	N/A<br>N/A
		Ethanol extract (percolation) (*A. tomentosum* trunk)	N/A	26.50 ± 3.50 µg/ml (W2)<br>25.00 ± 4.24 µg/ml (3D7)	N/A<br>N/A
		Ethanol extract (percolation) (*A. tomentosum* leaves)	N/A	23.75 ± 1.06 µg/ml (W2)<br>27.00 ± 5.66 µg/ml (3D7)	N/A<br>N/A
		Ethanol extract (percolation) (*A. tomentosum* fruits)	N/A	20.52 ± 1.41 µg/ml (W2)<br>38.55 ± 1.06 µg/ml (3D7)	N/A<br>N/A
		Ethanol extract (percolation) (*A. tomentosum* seeds)	N/A	24.51 ± 3.56 µg/ml (W2)<br>3.03 ± 0.20 µg/ml (3D7)	>20.4 (Vero)<br>>165.02 (Vero)
(63)	*P. falciparum*<br>W2 and 3D7	Ethanol extract	N/A	32.75 ± 1.06 µg/mL (W2)<br>20.51 ± 0.70 µg/mL (3D7)	>15.3 (Vero); >30.5 (HepG2)<br>>24.4 (Vero); >48.7 (HepG2)
		Neutral fraction	N/A	15.02 ± 2.83 µg/mL (W2)<br>17.75 ± 0.35 µg/mL (3D7)	29.9 (Vero); 16.7 (HepG2)<br>25.3 (Vero-3D7); 14.1 (HepG2-3D7)
		Alkaloid fraction	N/A	0.98 ± 0.20 µg/mL (W2)<br>7.63 ± 0.31 µg/mL (3D7)	305.8 (Vero); 75.9 (HepG2)<br>39.7 (Vero); 9.7 (HepG2)
		Uleine	N/A	0.75 ± 0.10 µg/mL (W2)<br>11.90 ± 0.10 µg/mL (3D7)	499.5 (Vero); 407.6 (HepG2)<br>31.5 (Vero); 25.3 (HepG2)

(64)	*P. falciparum*<br>K1 and W2	Methanol extract (A. leucostachyus aerial plant)<br>Methanol extract (A. leucostachyus aerial plant)<br>Methanol extract (A. leucostachyus aerial plant)<br>Ethanol extract (C. cajucara white variety bark)<br>Ethanol extract (C. cajucara white variety leaf)<br>Ethanol extract (C. cajucara red variety bark)<br>Ethanol extract (C. cajucara white variety bark)<br>Ethanol extract (C. cajucara white variety leaf)<br>Ethanol extract (C. cajucara red variety bark)<br>Ethanol extract (C. cajucara white variety bark)<br>Ethanol extract (C. cajucara white variety leaf)<br>Ethanol extract (C. cajucara red variety bark)<br>Chloroform extract (C. bullosa branch)<br>Chloroform extract (C. cajucara white variety leaf)<br>Chloroform extract (C. cajucara red variety leaf)<br>Chloroform extract (C. bullosa branch)<br>Chloroform extract (C. cajucara white variety leaf)<br>Chloroform extract (C. cajucara red variety leaf)<br>Chloroform extract (C. bullosa branch)<br>Chloroform extract (C. cajucara white variety leaf)<br>Chloroform extract (C. cajucara red variety leaf)	N/A	7.1 ± 3.3 µg/mL<br>13.3 ± 2.3 µg/mL<br>19.3 ± 5.5 µg/mL<br>17.2 ± 6.6 µg/mL<br>16.3 ± 4.5 µg/mL<br>32.2 ± 5.7 µg/mL<br>13.3 ± 2.0 µg/mL<br>10.2 ± 2.5 µg/mL<br>9.9 ± 3.2 µg/mL<br>10.5 ± 3.3 µg/mL<br>9.8 ± 1.8 µg/mL<br>15.6 ± 2.9 µg/mL<br>13.5 ± 2.7 µg/mL<br>11.3 ± 3.4 µg/mL<br>6.4 ± 1.2 µg/mL<br>47.4 ± 1.6 µg/mL<br>12.4 ± 4.1 µg/mL<br>19.3 ± 6.4 µg/mL<br>7.3 ± 1.8 µg/mL<br>19.5 ± 3.1 µg/mL<br>7.1 ± 3.3 µg/mL	>5 (J774)<br>>15.0 (J774)<br>3.3 (J774)<br>7.4 (J774)<br>>12.3 (J774)<br>>6.2 (J774)<br>3.5 (J774)<br>>19.6 (J774)<br>9.7 (J774)<br>>19.0 (J774)<br>0.7 (J774)<br>>12.8 (J774)<br>>14.8 (J774)<br>>17.7 (J774)<br>6.3 (J774)<br>>4.2 (J774)<br>5.7 (J774)<br>>10.4 (J774)<br>4.6 (J774)<br>1.5 (J774)<br>1.4 (J774)
(65)	*P. falciparum*<br>W2, Dd2	Andiroba oil<br>8.2 µg/ml (W2)<br>8.2 µg/ml (Dd2)<br>Limonoids-rich fraction<br>3.1 µg/ml (W2)<br>3.1 µg/ml (Dd2)	100% (W2)<br>31% (Dd2)<br><br>100% (W2)<br>56%	8.4 µg/ml (Dd2)<br><br>0.4 µg/ml (Dd2)	N/A<br>N/A<br><br>N/A<br>N/A

(66)	*P. falciparum* CQS and CQR	Aspidosperma indole alkaloid 1	N/A		
		CQR: 24h		16.3 ± 2.9 μM	N/A
		48h		3.8 ± 0.7 μM	N/A
		CQS: 24h		11.0 ± 1.7 μM	N/A
		48h		4.6 ± 0.5 μM	N/A
		Aspidosperma indole alkaloid 2	N/A		
		CQR: 24h		19.5 ± 7.2 μM	6.6 (NIH-3T3)
		48h		3.2 ± 0.9 μM	22.7 (NIH-3T3)
		CQS: 24h		13.1 μM	N/A
		48h		5.1 μM	N/A
		Aspidosperma indole alkaloid 3	N/A		
		CQR: 24h		16.1 ± 3.0 μM	7.9 (NIH-3T3)
		48h		5.6 ± 0.7 μM	15.6 (NIH-3T3)
		CQS: 24h		22.0 ± 7.1 μM	N/A
		48h		5.9 ± 1.5 μM	N/A
		Aspidosperma indole alkaloid 4	N/A		
		CQR: 24h		1.8 ± 0.9 μM	N/A
		48h		4.1 ± 0.6 μM	N/A
		CQS: 24h		9.3 ± 2.4 μM	N/A
		48h		6.6 ± 1.4 μM	N/A
		Aspidosperma indole alkaloid 5	N/A		
		CQR: 24h		22.3 ± 11.6 μM	2.3 (NIH-3T3)
		48h		5.6 ± 1.3 μM	8.3 (NIH-3T3)
		CQS: 24h		N/A	N/A
		48h		N/A	N/A
		Aspidosperma indole alkaloid 6	N/A		
		CQR: 24h		15.1 ± 1.9 μM	N/A
		48h		12.2 ± 5.2 μM	N/A
		CQS: 24h		21.5 ± 6.5 μM	N/A
		48h		20.3 ± 6.2 μM	N/A
		Aspidosperma indole alkaloid 7			
		CQR: 24h	N/A		
		48h		7.4 μM	N/A
				6.2 μM	N/A

		CQS: 24h			
		48h		34.0 µM	N/A
				15.4 µM	N/A
		Aspidosperma indole alkaloid 8			
		CQR: 24h	N/A		
		48h		15.4 ± 4.2 µM	2.1 (NIH-3T3)
				12.7 ± 4.2 µM	3.1 (NIH-3T3)
		CQS: 24h			
		48h		27.2 µM	N/A
				8.7 µM	N/A
		Aspidosperma indole alkaloid 9			
		CQR: 24h	N/A		
		48h		17.7 ± 4.9 µM	N/A
				28.5 ± 13.0 µM	N/A
		CQS: 24h			
		48h		40.8 ± 3.8 µM	N/A
				22.6 ± 2.5 µM	N/A
		Aspidosperma indole alkaloid 10			
		CQR: 24h	N/A		
		48h		52.8 ± 7.1 µM	N/A
				25.6 ± 2.7 µM	N/A
		CQS: 24h			
		48h		113.1 µM	N/A
				55.3 µM	N/A
		Aspidosperma indole alkaloid 11			
		CQR: 24h	N/A		
		48h		90.4 ± 43.7 µM	N/A
				59.2 ± 5.4 µM	N/A
		CQS: 24h			
		48h		44.4 µM	N/A
				28.0 µM	N/A
		Aspidosperma indole alkaloid 12			
		CQR: 24h	N/A		
		48h		149.7 ± 27.6 µM	0.1 (NIH-3T3)
				49.5 ± 3.7 µM	0.3 (NIH-3T3)
		CQS: 24h			
		48h		169.3 µM	N/A
				57.3 µM	N/A
(67)	*P. falciparum* K1	Essential oil (V. arborea)	N/A	7.00 ± 3.50 µg/mL	≥ 71.4 (HeLa/Mice macrophages)
		Essential oil (L. sidoides)		10.50 ± 2.80 µg/mL	45.7 (HeLa); 43.3 (Mice macrophages)
		Essential oil (C. zehntneri)		15.20 ± 3.30 µg/mL	≥ 32,8 (HeLa/Mice macrophages)
		Estragole		30.70 ± 3.10 µg/mL	≥ 16.3 (HeLa/Mice macrophages)
		Thymol		4.50 ± 1.70 µg/mL	≥ 111.1 (HeLa/Mice macrophages)
		α-Bisabolol		5.00 ± 2.30 µg/mL	≥ 100 (HeLa/Mice macrophages)

(68)	*P. falciparum* W2	Hydroethanolic extract	N/A	23.68 ± 3.08 µg/ml	> 10.56 (HepG2)
		Total alkaloids from the extract		9.93 ± 1.28 µg/ml	16.49 (HepG2)
		Total alkaloids from powder		15.82 ± 4.59 µg/ml	7.01 (HepG2)
		Total alkaloids from powder extract 1		12.78 ± 0.14 µg/ml	16.27 (HepG2)
		Total alkaloids from powder extract 2		8.75 ± 2.26 µg/ml	21.16 (HepG2)
		Total alkaloids from powder extract 3		34.17 ± 1.43 µg/ml	> 7.32 (HepG2)
		Alkaloid fraction by Stas-Otto-Gang 1		13.18 ± 1.20 µg/ml	20.10 (HepG2)
		Alkaloid fraction by Stas-Otto-Gang 4		12.03 ±0.48 µg/ml	23.01 (HepG2)
		Alkaloid fraction by Stas-Otto-Gang 7		18.30 ± 1.91 µg/ml	17.48 (HepG2)
		Alkaloid fraction by Stas-Otto-Gang 10		18.52 ± 0.60 µg/ml	18.07 (HepG2)
		Yohimbine		14,35 ± 2.77 µg/ml	11.77 (HepG2)
(70)	*P. falciparum* W2	Aqueous extract (S. cedron)	N/A	1.6 µg/mL	N/A
		Ethanol extract (A. rigidum)		6.0 ± 0.0 µg/mL	
		Dichloromethane extract (A. rigidum)		2.5 ± 0.7 µg/mL	
		Butanolic extract (A. rigidum)		2.5 ± 0.7 µg/mL	
		Aqueous extract (A. rigidum)		10.2 µg/mL	
		Ethanol extract (fruit peel) (B. excelsa)		4.5 ± 0.7 µg/mL	
		Ethanol extract (bark) (B. excelsa)		2.0 ± 0.0 µg/mL	
		Sap (bast) (B. excelsa)		7.5 ± 3.5 µg/mL	
		Ethanol extract (M. ferox)		20 µg/mL	
		Aqueous extract (A. amazonicus)		20 µg/mL	
		Ethanol extract (M. indica)		>50 µg/mL	
		Aqueous extract (O. hamiltonii)		>50 µg/mL	
		Seed oil (C. guianensis)		>50 µg/mL	
		Aqueous extract (U. guianensis)		>50 µg/mL	
		Ethanol extract (E. oleraceae)		>50 µg/mL	
		Aqueous extract (E. uchi)		>50 µg/mL	
(71)	*P. falciparum*	Limonoid 1	N/A	15.4 µM	>12 (MRC-5)
		Limonoid 2		15.3 µM	>13 (MRC-5)
		Limonoid 3		7.0 µM	>26 (MRC-5)
		Limonoid 4		20.7 µM	5 (MRC-5)
		Limonoid 5		5.0 µM	>44 (MRC-5)
(73)	*P. falciparum* K1 (MRA-159)	4-nerolidylcatechol	N/A	0.21 µg/mL	N/A
		4-nerolidylcatechol Derivative 2		11.14 µg/mL	
		4-nerolidylcatechol Derivative 3		1,56 µg/mL	
		4-nerolidylcatechol Derivative 4		1.15 µg/mL	
		4-nerolidylcatechol Derivative 5		0.35 µg/mL	
		4-nerolidylcatechol Derivative 6		Inactive	
		4-nerolidylcatechol Derivative 7		Partially Active	
		4-nerolidylcatechol Derivative 8		1.70 µg/mL	
		Catechol		8.88 µg/mL	
		Nerolidol		Partially Active	

(74)	*P. falciparum*<br>K1, 3D7, M1, M2	4-Nerolidylcatechol	N/A	0.60 ± 0.41 μg/mL (K1)<br>2.11 ± 1.15 μg/mL (3D7)<br>0.05 ± 0.06 μg/mL (M1)<br>0.83 ± 0.58 μg/mL (M2)	N/A
(75)	*P. falciparum*<br>W2	Physalin B<br>Physalin D<br>Physalin F<br>Physalin G	N/A	2.8 ± 1.20 μM<br>55 ± 0.96 μM<br>2.2 ± 1.16 μM<br>6.7 ± 0.37 μM	12.30 (Mouse splenocytes)<br>10.40 (Mouse splenocytes)<br>5.94 (Mouse splenocytes)<br>5.60 (Mouse splenocytes)
(76)	*P. falciparum* 3D7	Methyl 5,7-dimethoxy-2,2-dimethyl-<br>2H-1-benzopyran-6-propanoate	N/A	23.6 μM	>4.23 (KB)
(77)	*P. falciparum*<br>K1	Ethanol extract (leaf)<br>Chloroform fraction<br>Amplexine	N/A	35.8 μg/mL<br>10.5 μg/mL<br>7.1 μg/mL	>1.39 (MCR-5)<br>>4.76 (MCR-5)<br>>7.04 (MCR-5)
(78)	*P. falciparum*<br>W2 and 3D7	Volatile oil	N/A	1.21 ± 0.05 μg/mL (W2)<br>2.30 ± 0.09 μg/mL (3D7)	>80 (W2)<br>>40 (3D7)
(79)	*P. falciparum*<br>W2	Piplartine<br>1b<br>1g<br>1k<br>1m<br>14f<br>Piperine	N/A	20.54 μg/ml<br>>200 μg/ml<br>>200 μg/ml<br>>200 μg/ml<br>>104.45 μg/ml<br>>200 μg/ml<br>>200 μg/ml	0.73 (HepG2); 1.14 μg/mL (Vero)<br>N/A<br>N/A<br>N/A<br>> 4.79 (HepG2); > 4.79 (Vero)<br>N/A<br>N/A
(80)	*P. falciparum*<br>K1	3,4,5,6-tetradehydro-β-yohimbine<br>19E-hunteracine<br>20-epi-dasycarpidone<br>20(E)-nor-subincanadine E<br>12-hydroxy-N-acetyl-21(N)-dehydroplumeran-18-oic acid	N/A	14.0 ± 2.7 μg/ml<br>> 50.0 μg/ml<br>4.5 ± 0.2 μg/ml<br>14.5 ± 2.8 μg/ml<br>> 50.0 μg/ml	>3.57 (NIH-3T3)<br><br>>11.11 (NIH-3T3)<br>>3.44 (NIH-3T3)
(81)	*P. falciparum*<br>W2	Tormentic acid	N/A	11.4 μg/mL	N/A
(83)	*P. falciparum*<br>W2	Dichloromethane fraction	N/A	22.9 ± 0.2 μg/mL	N/A
(84)	*P. falciparum* 3D7	Ethanol extract<br>Dichloromethane fraction<br>Ethyl acetate fraction<br>Methanol fraction<br>Eleutherol<br>Eleutherine<br>Isoeleutherine<br>S2 fraction (eleutherin + isoleutherin)	N/A	55.65 ± 9.34 μg/mL<br>81.94 ± 32.08 μg/mL<br>10.22 ± 2.38 μg/mL<br>89.91 ± 29.67 μg/mL<br>>200 μg/mL<br>10.45 ± 3.13 μg/mL<br>8.70 ± 2.45 μg/mL<br>3.67 ± 1.01 μg/mL	N/A
(85)	*P. falciparum*<br>W2	Ethanol extract<br>FrA<br>FrB<br>BP-181-7<br>BP204 (caryatin)	N/A	4.8 ± 2.1 μg/ml<br>2.5 ± 1.2 μg/ml<br>26 ± 10 μg/ml<br>7.2 μg/ml<br>3.5 ± 1.3 μg/ml	8 (BGM)<br>22 (BGM)<br>6.82 (BGM)<br>≥35 (BGM)<br>≥286 (BGM)

*Cell lines used for selectivity index (SI) analysis are provided when applicable. # quantification of parasitaemia reduction through the [3H]-hypoxanthine uptake

assay; ## quantification of parasitaemia reduction through the histidine-rich protein 2 (HRPII) assay. MPM: mouse peritoneal macrophages; CQS - Chloroquine susceptible; CQR - chloroquine resistant; N/A: non applicable.

TABLE V

In vivo aspects of the active compounds of the studied Brazilian plants

Reference	*Plasmodium* strain	Mice	Dosage (mg/kg/d) and route of administration	Blood smear	Treatment regimen	Tested compound	% Parasitaemia reduction and dosage (mg/kg)
(16)	*P. berghei* NK65	Swiss	500 (oral)	Day 5	4 days	*H. reniformis* roots ethanol extract)	67%
						H. reniformis Leaves ethanol extract)	48%
(17)	*P. berghei* NK65	Swiss	250 and 500 (oral)	Day 10	3 days	*A. ramiflorum* neutral precipitate	66% at 250 mg/kg
							53% at 500 mg/kg
						A. ramiflorum nonsoluble	16% at 250 mg/kg
						C. maxima seed ethanol extract	22% 500 mg/kg
						No significant parasitaemia reduction in *M. charantia* groups	
(20)	*P. berghei* ANKA	Swiss	100 and 200 (oral)	Days 5 to 10	3 days	*A. olivaceum* acidic fractions	79% at 100 mg/kg
							58% at 200 mg/kg
(22)	*P. berghei* ANKA	BALB/c	10, 100, and 200 (oral)	Day 11	8 days	*C. reticulada* oleoresin	96% at 200 mg/kg
							93% at 100 mg/kg
							11% at 10 mg/kg
(23)	*P. berghei* NK65	Swiss	100 (oral)	Days 5 and 10	3 days	*A. parvifolium* root-bark ethanol extract	79 and 29%
						A. parvifolium root ethanol extract	75 and 52%
						A. parvifolium bark hydromethanolic fraction	93 and 57%

						A. parvifolium bark chloroform fraction	79 and 57%
(24)	*P. chabaudi*	C57BL/6	20, 30 and 40 (oral)	Days 3 to 15	13 days	*E. oleracea* total phenolics	89.4 and 77.3% at 20 mg/kg (days 6 and 7)
							81 and 62.2% at 15 mg/kg (days 6 and 7)
							54.2% at 10 mg/kg (day 6)
(25)	*P. chabaudi*	C57BL/6	50 and 25 (intraperitoneal)	Days 3 to 8	4 days	*C. pluviosa* 50% ethanolic fraction	79.4 and 72.1% at 50 mg/kg (days 6 and 7)
(45)	*P. berghei* NK65	C57BL/6	250, 500 and 1000 (oral)	Days 5, 7 and 9	3 days	*A. campestris* ethanol extract	42.1, 37, and 10.9% at 1000 mg/kg (days 5,7 and 9)
(46)	*P. berghei* NK65	N/A	500 and 1000 (oral)	Day 5	4 days	*B. pilosa* whole-plant extract	40%
(48)	*P. berghei* NK65	Swiss	125 and 250 (oral)	Days 5 and 7	4 days	*A. nitidum* ethanol extract	48 and 49% at 125 mg/kg
							19 and 33% at 125 mg/kg
						A. nitidum chloroform fraction	43 and 63% at 250mg/kg
							14 and 27% at 125 mg/kg
						A. nitidum ethyl acetate fraction	14 and 27% at 125 mg/kg
							24 and 39% at 250 mg/kg
(51)	*P. berghei* NK65	Swiss	500 and 1000 (oral)	Day 5	4 days	*R. ferruginea* extract	48% at 1000 mg/kg
						R. ferruginea extract	34% at 500 mg/kg
(52)	*P. berghei*	Swiss	250, 500 and	Days 5	4 days	*B. pilosa* extract from Belo Horizonte	38 and 44% at 250 mg/kg

	NK65		1000 (oral)	and 7			55 and 40% at 500 mg/kg
							54 and 60% at 1000 mg/kg
						B. pilosa extract from Ibiá	30 and 48% at 250 mg/kg
							43 and 38% at 500 mg/kg
							0 and 10% at 1000 mg/kg
						B. pilosa extract from Montes Claros	55 and 60% at 250 mg/kg
							32 and 36% at 500 mg/kg
							17 and 24% at 1000 mg/kg
(54)	*P. berghei* NK65	Swiss	100 and 200 (oral)	Days 5 and 7	3 days	No significant parasitaemia reduction	
(58)	*P. berghei*	Swiss	1000 (oral)	Day 5	4 days	*A. australe* leaves extract	40%
						E. febriburga stalk bark extract)	43%
						L. speciosus roots extract	38%
						T. guianensis root extract	39%
(59)	*P. berghei* ANKA	BALB/c	5 (oral)	Days 6, 9 and 11	5, 7 and 9 days	No significant parasitaemia reduction	
(64)	*P. berghei* NK65	BALB/c	250 (oral)	Days 5 and 7	4 days	*A. leucostachyus* aerial plant methanol extract	1 and 48%
						C. cajucara leaf chloroform extract	19 and 0%
						X. amazonica leaf chloroform extract	52 and 11%
(67)	*P. berghei* NK65	Swiss	100, 500, and 1000 (oral)	Days 5 and 7	4 days	*V. arborea* ethanol extract (subcutaneous)	40 and 47% at 500 mg/kg
						V. arborea ethanol extract (oral)	20 and 0% at 1000 mg/kg
						L. sidoides ethanol extract (oral)	55 and 45% at 100 mg/kg

			25, 50, 100, and 500 (subcutaneous)			*C. zehntneri* ethanol extract (oral)	53.5 and 43% at 500 mg/kg
(69)	*P. berghei*	Swiss	250 (oral)	Days 5 and 7	4 days	*B. pilosa* ethanol extract	36% and 29%
						B. pilosa ether: methanol fraction	38% and 0%
(71)	*P. berghei* NK65	BALB/c	50 and 100 (oral)<br><br>50 and 100 (subcutaneous)	Days 5 and 7	4 days	Limonoid 3 (oral)	65.7 and 46.3% at 100 mg/kg
						Limonoid 3 (subcutaneous)	44.2 and 30.4% at 100 mg/kg
						Limonoid 4 (oral)	40.3 and 28.9% at 100 mg/kg
						Limonoid 4 (subcutaneous)	38.6 and 21.7% at 100 mg/kg
(72)	*P. berghei* NK65	N/A	12.5 (intraperitoneal)	Days 5 and 7	4 days	*A. crassiflora* hexanic fraction	0 and 25%
						A. crassiflora chloroformic I fraction	60 and 52%
						A. crassiflora chloroformic II fraction	0 and 28%
(74)	*P. berghei* NK65	Swiss	200, 400, and 600 (oral and subcutaneous) 200 (intraperitoneal)	Days 5 and 7	4 days	4-Nerolidylcatechol (4-NC)	63.1 and 59.7% at 600 mg/kg (oral)
							34.4 and 48.8% at 400 mg/kg (oral)
							0 and 61.3% at 600 mg/kg (subcutaneous)
							0 and 40.6% at 400 mg/kg (subcutaneous)
(75)	*P. berghei* NK65	BALB/c	50 and 100 (intraperitoneal)	Days 4 to 8	4 days	Physalin D	65% at 100 mg/kg (day 8)
(77)	*P. berghei* NK65	Swiss	500 (subcutaneous)	Days 5 and 7	4 days	*Tachia sp.* water extract from root	59 and 24%
						Tachia sp. methanol extract from root	39 and 11%

			500 (oral)		4 days	*Tachia sp.* methanol extract from leaf	24 and 0%
(78)	*P. berghei*	BALB/c	10, 100, and 200 (oral)	Day 11	8 days after 4 days of infection	*C. articulatus* volatile oil	75.2% at 200 mg/kg
							74.8% at 100 mg/kg
							48.9% at 10 mg/kg
(81)	*P. berghei* NK65	Swiss	10 and 15 (oral)	Day 8	4 days	Tormentic acid	58% at 15 mg/kg
							35% at 10 mg/kg
						b-Sitosterol	51% at 15 mg/kg
							20% at 10 mg/kg
(82)	*P. berghei*	Swiss	1000 (oral)	Day 9	5 days	No significant parasitaemia reduction	
(83)	*P. berghei* ANKA	Swiss	200 (oral)	Days 1 to 5	10 consecutive days (pre-infection)<br>5 consecutive days (post-infection)	*H. articulatus* ethanol extract	35.4%
(85)	*P. berghei* NK65	Swiss	100 and 200 (oral)	Days 5 and 7	3 days	No significant parasitaemia reduction	
